# Secondary resistance to anti-EGFR therapy by transcriptional reprogramming in patient-derived colorectal cancer models

**DOI:** 10.1186/s13073-021-00926-7

**Published:** 2021-07-16

**Authors:** Deepak Vangala, Swetlana Ladigan, Sven T. Liffers, Soha Noseir, Abdelouahid Maghnouj, Tina-Maria Götze, Berlinda Verdoodt, Susanne Klein-Scory, Laura Godfrey, Martina K. Zowada, Mario Huerta, Daniel L. Edelstein, Jaime Martinez de Villarreal, Miriam Marqués, Jörg Kumbrink, Andreas Jung, Tobias Schiergens, Jens Werner, Volker Heinemann, Sebastian Stintzing, Doris Lindoerfer, Ulrich Mansmann, Michael Pohl, Christian Teschendorf, Christiane Bernhardt, Heiner Wolters, Josef Stern, Selami Usta, Richard Viebahn, Jacob Admard, Nicolas Casadei, Stefan Fröhling, Claudia R. Ball, Jens T. Siveke, Hanno Glimm, Andrea Tannapfel, Wolff Schmiegel, Stephan A. Hahn

**Affiliations:** 1 Department of Molecular GI Oncology, Faculty of Medicine, Ruhr University Bochum, 44780 Bochum, Germany; 2 Department of Internal Medicine, Ruhr University Bochum, Knappschaftskrankenhaus, Bochum, Germany; 3 Institute of Pathology, Ruhr University of Bochum, Bochum, Germany; 4 Present Address Division of Solid Tumor Translational Oncology, West German Cancer Center, University Hospital Essen, Essen, Germany; 5 Bridge Institute of Experimental Tumor Therapy, West German Cancer Center, University Hospital Essen, Essen, Germany; 6 Division of Solid Tumor Translational Oncology, German Cancer Consortium (DKTK, partner site Essen) and German Cancer Research Center, DKFZ, Heidelberg, Germany; 7 Translational Functional Cancer Genomics, NCT Heidelberg and German Cancer Research Center (DKFZ), Heidelberg, Germany; 8 Faculty of Biosciences, Heidelberg University, Heidelberg, Germany; 9 Department of Translational Medical Oncology, National Center for Tumor Diseases (NCT), Dresden, and German Cancer Research Center (DKFZ), Dresden, Germany; 10 Medical Scientific Affairs, Sysmex Inostics Inc., Baltimore, MD USA; 11 Epithelial Carcinogenesis Group, Spanish National Cancer Research Centre (CNIO) and CIBERONC, Madrid, Spain; 12 Institute of Pathology, Ludwig Maximilian University (LMU), Munich, Germany; 13 German Cancer Consortium (DKTK, partner site Munich), Munich, Germany; 14 Department of General, Visceral, and Transplantation Surgery, University Hospital, LMU Munich, Munich, Germany; 15 Department of Medicine III, University Hospital, LMU Munich, Munich, Germany; 16 Department of Hematology, Oncology, and Tumor Immunology (CCM) Charité Universitaetsmedizin Berlin, Berlin, Germany; 17 Institute for Medical Information Processing, Biometry and Epidemiology, Ludwig-Maximilians-Universität München, Munich, Germany; 18 Department of Internal Medicine, St. Josefs-Hospital, Dortmund, Germany; 19 Department of Visceral and General Surgery, St. Josef Hospital, Dortmund, Germany; 20 Department of Surgery, Ruhr University Bochum, Knappschaftskrankenhaus, Bochum, Germany; 21 Institute of Medical Genetics and Applied Genomics, University of Tübingen, Tübingen, Germany; 22 German Cancer Consortium (DKTK), Heidelberg, Germany; 23 Deptartment of Translational Medical Oncology, NCT Heidelberg and German Cancer Research Center, Heidelberg, Germany; 24 Center for Personalized Oncology, NCT Dresden and University Hospital Carl Gustav Carus Dresden at TU Dresden, Dresden, Germany; 25 German Cancer Consortium (DKTK), Dresden, Germany

**Keywords:** Anti-EGFR, PDX, Secondary resistance, Targeted treatment, Transcriptional reprogramming

## Abstract

**Background:**

The development of secondary resistance (SR) in metastatic colorectal cancer (mCRC) treated with anti-epidermal growth factor receptor (anti-EGFR) antibodies is not fully understood at the molecular level. Here we tested in vivo selection of anti-EGFR SR tumors in CRC patient-derived xenograft (PDX) models as a strategy for a molecular dissection of SR mechanisms.

**Methods:**

We analyzed 21 *KRAS, NRAS, BRAF,* and *PI3K* wildtype CRC patient-derived xenograft (PDX) models for their anti-EGFR sensitivity. Furthermore, 31 anti-EGFR SR tumors were generated via chronic in vivo treatment with cetuximab. A multi-omics approach was employed to address molecular primary and secondary resistance mechanisms. Gene set enrichment analyses were used to uncover SR pathways. Targeted therapy of SR PDX models was applied to validate selected SR pathways.

**Results:**

In vivo anti-EGFR SR could be established with high efficiency. Chronic anti-EGFR treatment of CRC PDX tumors induced parallel evolution of multiple resistant lesions with independent molecular SR mechanisms. Mutations in driver genes explained SR development in a subgroup of CRC PDX models, only. Transcriptional reprogramming inducing anti-EGFR SR was discovered as a common mechanism in CRC PDX models frequently leading to RAS signaling pathway activation. We identified cAMP and STAT3 signaling activation, as well as paracrine and autocrine signaling via growth factors as novel anti-EGFR secondary resistance mechanisms. Secondary resistant xenograft tumors could successfully be treated by addressing identified transcriptional changes by tailored targeted therapies.

**Conclusions:**

Our study demonstrates that SR PDX tumors provide a unique platform to study molecular SR mechanisms and allow testing of multiple treatments for efficient targeting of SR mechanisms, not possible in the patient. Importantly, it suggests that the development of anti-EGFR tolerant cells via transcriptional reprogramming as a cause of anti-EGFR SR in CRC is likely more prevalent than previously anticipated. It emphasizes the need for analyses of SR tumor tissues at a multi-omics level for a comprehensive molecular understanding of anti-EGFR SR in CRC.

**Supplementary Information:**

The online version contains supplementary material available at 10.1186/s13073-021-00926-7.

## Background

The addition of epidermal growth factor receptor (EGFR) targeting monoclonal antibodies (mABs) cetuximab and panitumumab to treatment protocols has improved survival rates in patients with metastatic colorectal cancer (CRC) as has been shown in numerous clinical trials [1, 2]. An early finding in the treatment of CRC patients with anti-EGFR targeting antibodies was that only a subgroup benefitted from treatment. Clinical trials report a median overall survival benefit of approximately 8 months for CRC without *KRAS* and *NRAS* mutations for anti-EGFR antibody-containing regimens [3]. Despite initial clinical benefit from treatment with anti-EGFR mABs, virtually all patients acquire resistance to anti-EGFR therapy within 10 to 12 months, eventually leading to disease progression.

Currently, the acquisition of tissue and subsequent genotyping of metastatic CRC lesions with suspected acquired or secondary resistance (SR) to anti-EGFR antibodies presents a challenge even in the most advanced clinical practice and is not routinely performed in clinical workup at disease progression. Therefore, molecular mechanisms of acquired SR to anti-EGFR have been primarily addressed by cell culture models, in a limited number of tissue biopsies from cetuximab (CET)-treated patients and very few patient-derived xenograft (PDX) models [4–14]. By far the largest fraction of the current molecular data on SR was generated indirectly by analyzing the emergence of mutated circulating tumor DNA (ctDNA) in the blood of CRC patients under anti-EGFR treatment. This led, apart from the identification of *FGFR1*, *ERBB2, NRG, GNAS,* and *MET* amplifications, to the discovery of emerging *KRAS*, *NRAS*, *PIK3CA*, *BRAF, EGFR,* and *IRS1* mutations in resistant CRC subpopulations mediating SR towards anti-EGFR therapy [4, 6, 7, 12, 13, 15–18]. However, several contradictory reports on the prevalence of SR driver mutations derived from primary CRC tissue appeared in recent years [4–8, 11–14]. Furthermore, the emergence of *RAS* mutation*s* in ctDNA at the time of disease progression in patients undergoing anti-EGFR therapy was reported to be neither associated with a shorter progression-free survival nor predictive for any cytoreduction [8, 19]. This suggests that as tumors undergo clonal expansion in response to targeted therapy, not every mutation identified in ctDNA may be driving SR and that other molecular mechanisms may play a role in acquiring resistance to anti-EGFR antibodies. Furthermore, mathematical modeling of the dynamic of resistance development in PDXs under vertical blockade of the EGFR pathway by Misale et al. concluded that their data supported a model which beyond point mutations in driver genes other non-genetic or genetic mechanisms are required to achieve the high number of cetuximab-resistant cells at the start of treatment [10].

Here we asked the question, whether in vivo selection of anti-EGFR SR tumors from multiple CRC-based PDX models is an efficient strategy to generate broad collections of SR PDX tumors. The availability of such collections would provide an abundant source of tissues from SR PDX tumors to allow in-depth molecular examinations that are currently not feasible to perform from on-treatment patient tissue biopsies. Furthermore, comprehensive pre-testing of next-generation compounds and various targeted therapy strategies aimed at overcoming SR mechanisms would greatly improve the identification of promising therapeutic combinations to be considered for clinical evaluation.

## Methods

### PDX biobank and treatment cohorts

Tissue samples to establish the colon cancer PDX bank were collected from 154 patients following surgical intervention for colon cancer at the Ruhr University Comprehensive Cancer Center or the Comprehensive Cancer Center of the Ludwig-Maximilians University Munich. Each PDX model derived from an individual patient tumor received a unique identification number (ID), preceded by the letters “BoC” (“Bo” for Bochum as the site where the PDX tumor was established, and “C” for colorectal carcinoma). The ID is then followed by one of the three-letter codes indicating the treatment cohort the individual mouse was randomly assigned to (K, for untreated control; 5dC for mice treated for 5 days with cetuximab; C for mice chronically treated with cetuximab) followed by another ID number for each of the two implantation sites and tumors potentially growing per site and mouse. From the PDX bank, we randomly selected 29 PDX models representing 29 individual patient’s tumors for the treatment study reported herein. A total of 21 PDX models with wildtype sequences based on targeted NGS sequencing in the genes *KRAS, NRAS, PIK3CA*, and *BRAF* (therefore called quadruple wildtype) as well as 8 PDX models harboring an activating mutation in one of the aforementioned genes were selected. Informed and written consent was obtained from all patients. The study was approved by the Ruhr University Bochum Ethics Committee, Registry no. 3841-10 & 16-5792, and the Ludwig-Maximilians University Munich Ethics Committee, project no. 131-16. All animal experiments were approved by the local authorities (84-02.04.2012.A360 & 84-02.04.2015.A135) and performed in accordance with the guidelines for Ethical Conduct in the Care and Use of Animals.

To establish treatment cohorts, tumor pieces (1–2 mm) from early passage PDXs (≤ F4 generation) were soaked in undiluted matrigel (Becton Dickinson) for 15 to 30 min and subsequently implanted subcutaneously onto 5- to 10-week-old female mice (NMRI-*Foxn1nu/Foxn1nu*, Janvier, St Berthevin Cedex, France) at two sites (scapular region) using as many as 4 pieces per site. Tumors were allowed to grow to a size of approx. 100–200 mm^3^, at which time mice were randomized in the treatment and control groups with five to six mice in each group. Tumor volumes were estimated from 2-dimensional tumor measurements by bi-weekly caliper measurements during the first 21 days and weekly measurements thereafter using the following formula: tumor volume (mm^3^) = [length (mm) × width (mm)^2^]/2. Complete response (CR) was defined as an undetectable tumor by macroscopical inspection and partial response (PR) by at least a 30% reduction in mean tumor volume compared to the mean tumor volume at the start of treatment. Disease progression was defined as a more than 20% increase in mean tumor volume determined at least at two consecutive time points compared to the tumor volume at the beginning of treatment. All other measurements were defined as stable disease. Growth curves were established by determining mean tumor volumes at different time points relative to the mean tumor volume at treatment start. For BoC106, time to progression (20% increase in mean tumor volume) was calculated relative to the mean tumor volume at week 24 (re-initiation of CET treatment). The identity of corresponding PDX tumors was secured via STR analyses using the GenomeLab Human STR Primer Set (Beckman Coulter, Krefeld, Germany) according to the manufacturer’s protocol. STR reactions were run on a CEQ8800 sequencer (Beckman Coulter). Mice were treated with CET (Merck KGaA) by intraperitoneal (i.p.) injection twice per week and dosed at 25 mg/kg, rolipram (Hycultec) daily i.p. at 1.6 mg/kg, vorinostat (Hycultec) daily i.p. at 25 mg/kg, refametinib (Selleckchem) daily by oral gavage at 25 mg/kg, ruxolitinib (Hycultec) twice daily by oral gavage at 180 mg/kg, Ly2874455 (MedKoo Biosciences) twice daily by oral gavage at 3 mg/kg, SCH772984 (MedChemTronica ) twice per day i.p. for five times per week with two consecutive days of treatment pause at 12.5 mg/kg, and trametinib (MedKoo Biosciences) five times per week with two consecutive days of treatment pause by oral gavage dosed at 0.5 mg/kg.

### Cell lines

BoC20 was derived via the standard outgrow method. The identity of BoC20 was confirmed by comparing STR data between the BoC20 PDX founder tumor and the derived primary cell line. DiFi cells were provided by P. Bastiaens, Systemic Cell Biology, Max Planck Institute of Molecular Physiology, Dortmund, Germany. The authenticity of DiFi cells was confirmed by STR profiling and comparison to the STR data in the Expasy database [20] (https://web.expasy.org/cellosaurus/CVCL_6895). STR analyses were performed as described by Dirks W.G. et al. [21] and analyzed on a CEQ8800 sequencer (Beckman Coulter). BoC20 cells were cultured in DMEM/DMEM-F12 medium (Thermo Fisher Scientific Inc.) supplemented with Rock-Inhibitor Y-27632 (Selleckchem) and 5% fetal calf serum (FCS) and penicillin/streptomycin (Thermo Fisher Scientific Inc.). DiFi cells were cultured in standard DMEM supplemented with penicillin/streptomycin, L-glutamine, sodium pyruvate, and 10% FCS (Thermo Fisher Scientific Inc.). Cells were maintained in a humidified incubator with 5% CO_2_ at 37 °C. BoC20 and DiFi cells were confirmed negative for mycoplasma by routine testing.

### Targeted NGS

Two hundred fifty nanograms genomic DNA was used per sample to produce sequencing libraries with the TruSeq Amplicon Cancer Panel (Illumina) according to the manufacturer’s protocol. The chosen panel covered 48 cancer-related genes (Additional file 1: Table S1), interrogating among others the known mutations in genes involved in primary and secondary anti-EGFR resistance. The 212 amplicons were simultaneously amplified in a single tube reaction. Briefly, the regions of interest were enriched by hybridizing sequence-specific oligonucleotides to the genomic DNA followed by ligation extension of the bounded oligos. The marked regions were further amplified by PCR with primers containing index barcodes for sample multiplexing. Finally, libraries were normalized by bead normalization prior to sequencing. Pooled libraries were sequenced on a MiSeq instrument (Illumina) using 2 × 150 bp paired-end reads. For data processing, fastq files were analyzed with the NextGENe V2.3.4 (SoftGenetics) software. For variant calling, raw reads were aligned to the human hg 19 assembly and primer sequences were soft-clipped prior to variant calling. Variants with a minor AF of ≥ 5% within the coding region and a minimum coverage of 10 variant reads were considered as alteration and visually confirmed with NextGENe.

### Exome sequencing

DNA quality was assessed on the Agilent TapeStation System (Agilent Technologies, Inc., Santa Clara, USA). Illumina libraries were constructed with SureSelect Human All Exon V7 (Agilent Technologies, Inc.) enrichment panel following the manufacturer’s instructions. All libraries were sequenced on the Illumina NovaSeq 6000 device in paired-end mode with 2 × 101 bp reads targeting an average coverage of > 200×. Reads were preprocessed with SeqPurge [22] (version 2019_05) using default parameters to remove adapter contamination and low-quality bases. For read alignment, a graft genome was constructed by concatenating the human reference genome (build GRCh37) and the mouse reference genome (build GRCm38p6). Alignment to the graft genome was performed with BWA-MEM [23] (version 0.7.17; https://arxiv.org/abs/1303.3997). Alignments were then reduced to human chromosomes only and with mapping quality of at least q = 30, in addition alignments filtered to allow a maximum of 3 mismatches and 1 insertion or deletion per alignment. Alignment quality was analyzed using MappingQC [24] (version 2019_05). FreeBayes [25] (https://arxiv.org/abs/1207.3907) was used to call variants on the filtered alignment data, with an AF cut-off of 5%. The remaining variant calls were annotated with Ensembl Variant Effect Predictor [26] to assess variant impact and to add population frequencies from gnomAD, ExAC, and 1000 Genomes databases [27–29] and variants with a population frequency of more than 1% in any of the databases were discarded. Mutation significance was assessed with Cancer Genome Interpreter [30] (https://www.cancergenomeinterpreter.org/home), further annotating alterations as known or predicted cancer drivers. Only known and predicted (tier 1) drivers are reported as likely pathogenic. Tier 2 predicted drivers are reported as of unclear significance. Exome sequencing has been deposited into the NCBI BioProject database under the BioProject ID PRJNA596887 (https://www.ncbi.nlm.nih.gov/bioproject/?term=PRJNA596887) [31].

### RNA sequencing

RNA quality was assessed with the Agilent 2100 Bioanalyzer RNA Nano kit and Agilent Fragment Analyzer total RNA kit (Agilent Technologies, Inc., Santa Clara, USA). Samples with RNA integrity number > 7 were selected for library construction. QuantSeq 3′ mRNA-Seq Library Prep Kit FWD UMI (Lexogen, Greenland, USA) was used with 100 ng of total RNA input to construct Illumina libraries according to the manufacturer’s manual. Libraries were sequenced on Illumina NextSeq 550 platform in single-end mode with 150 bp reads targeting 10 million reads per sample. Library preparation and sequencing procedures were performed by the same individual, and a design aimed to minimize technical batch effects was chosen. Quality of raw RNA-seq data in FASTQ files was evaluated to identify potential sequencing cycles with low average quality and base distribution bias and reads were then preprocessed using fastp [32] (version 0.20.0). Cleaned reads were aligned using STAR [33] (version 2.7.3a) allowing spliced read alignment to a graft genome containing human and mouse chromosomes (see DNA). Alignment quality was analyzed using MappingQC [24] (version 2019_08) and visually inspected with Broad Integrative Genome Viewer [34] (version 2.7.2). Alignments were collapsed based on the unique molecular identifier sequence (UMI) with UMI-tools [35] (version 1.0.0) to obtain one alignment per original fragment. Based on the Ensembl genome annotation (GRCh37 and GRCm38, Ensembl release 97) and using only uniquely aligned reads across the whole grafted reference, read counts for all human and mouse genes were obtained using subread [36] (version 2.0.0). The RNA sequencing data from our study have been deposited in the NCBI’s Gene Expression Omnibus (GEO) database (accession number GSE141861; https://www.ncbi.nlm.nih.gov/geo/query/acc.cgi?acc=GSE141861) [37].

### Copy number variation analysis

For BoC10 C3 & BoC20 C9, copy number variation (CNV) data were extracted from an independently generated set of exome sequencing data. DNA was isolated from the SR PDX tumors BoC10 C3 and BoC20 C9, respectively, as well as from one untreated control PDX tumor each (BoC10 K14 and BoC20 K11), and from corresponding patient normal tissue of both PDX models using the DNeasyBlood & Tissue Kit or QIAamp DNA MiniKit (Qiagen), and DNA concentrations were assessed by QubitTM dsDNABR AssayKit500 (Life Technologies). Tissue authentication was performed using the Investigator ESSplex Plus Kit (Qiagen), and DNA quality was verified using an Agilent2200 TapeStation. Library preparation was performed on an Agilent NGS Workstation (version F.0) using the SureSelectXT Automation Reagent Kit (Agilent) and Human All Exon V5 + UTRs reagents (Agilent). Paired-end sequencing (2 × 100 bp) was done on a HiSeq2000 applying TruSeqPEClusterKitv3 and TruSeqSBSKitv3 (Illumina). Paired-end DNA sequencing reads were mapped to a concatenated human and murine genome assembly to reduce false positive mutations introduced by murine contamination in the xenograft samples (1000 genomes [29] phase 2 reference assembly (hs37d5) (ftp://ftp.1000genomes.ebi.ac.uk/vol1/ftp/technical/reference/phase2_reference_assembly_sequence/) and the UCSC mm10 genome assembly [38] (http://hgdownload.cse.ucsc.edu/goldenPath/mm10/bigZips/chromFa.tar.gz)). Reads were aligned using BWA [23] (version 0.6.2-r126-tpx) allowing for soft clipping of bases of up to Phred score Q20 with a Q20 Phred score quality threshold for read trimming down to 35 bp (-q 20). Subsequent alignments were merged using samtools [39] (version 0.1.19-44428 cd), and PCR duplicates were marked using Picard tools [40] (version 1.90). Exome sequencing has been deposited into the European Genome-phenome Archive (EGA) database under the EGA study ID EGAS00001005320 (https://ega-archive.org/studies/EGAS00001005320) [41]. VarScan2 [42] was used to identify somatic CNAs in the exome samples (with the preceding sample used as the reference control) using the recommended workflow. Regions were filtered for unmappable genomic stretches and merged by requiring at least 70 markers per called copy number event. We selected regions with a log ratio of tumor coverage over control coverage higher than 0.55 or lower than − 0.55 as copy number gains and losses, respectively, and annotated them with RefSeq genes using BEDTools [43]. We searched for structural variants such as translocations that might lead to gene fusions with CREST [44] on the DNA level.

For BoC35 C1, BoC60 C5, BoC69 C5, and BoC106 C2, the SNPs (single-nucleotide polymorphisms) represented on the Infinium® MethylationEPIC BeadChip Array were used for CNV analyses. Un-normalized probe signal intensities were extracted from the ChAMP Bioconductor package [45] after default filtering and removal of potential mouse contaminated probes. Therefore, mouse tail DNA was hybridized to the EPIC array and 325,107 probes that passed a detection p value cut-off of 0.01 were excluded. Preprocessed data were imported into Partek Genomic Suite (version 7.18.0130). SR sample intensities were normalized to their corresponding 5-day CET treated controls, respectively. After log2 transformation copy numbers were adjusted and genomic copy number segmentation was performed. Different from default settings, a minimum of 50 genomic markers was used, while gender information was ignored. A copy number < 1 was termed as deleted, and an amplification had to exhibit a copy number > 3.

### Gene expression analyses and data processing.

An amount of 100 ng of every total RNA sample was hybridized to Agilent whole-genome expression microarrays (Human GE 4x44K, v2 G4845A, AMADID 026652, Agilent Technologies). RNA labeling, hybridization, and washing were carried out according to the manufacturer’s instructions. Images of hybridized microarrays were acquired with a DNA microarray scanner (Agilent G2505B), and features were extracted using the Agilent Feature Extraction image analysis software (AFE) version A.10.7.3.1 with default protocols and settings. The AFE algorithm generates a single intensity measure for each feature, referred to as the total gene signal (TGS), which was used for further data analyses using the GeneSpring GX software package version 14.9.1. AFE - TGS were normalized by the quantile method. Subsequently, data were filtered on normalized expression values. The gene expression data from our study have been deposited in the NCBI’s Gene Expression Omnibus (GEO) database (accession number GSE140973; https://www.ncbi.nlm.nih.gov/geo/query/acc.cgi?acc=GSE140973) [46].

### Vectors and site-directed mutagenesis

The coding sequence of *PPP2R5A* was inserted into the pSFFV-d4EGFP vector (gift from L. Naldini, [47]) by replacing the d4GFP coding sequence. Site-directed mutagenesis was performed using QuikChange II XL (Agilent) and custom-designed primers (PPP2R5A-R112L-mut-s: TGGTTGAGTATGTTTCAACTAATCTTGG-TGTAATTGTTGAATCAG and PPP2R5A-R112L-mut-as: CTGATTCAACAATTACACCAAGATTAGTTGAAACAT-ACTCAACCAG) to generate the *PPP2R5A* R112L mutant. The full-length sequence of each vector was assessed using Sanger sequencing to confirm the presence of the wildtype or the intended mutation and that no other mutations had been inserted.

### Transfection and transduction

HEK293T cells were transfected with pSFFV-d4EGFP (control), pSFFV-*PPP2R5A*, or pSFFV-PPP2R5A-R112L lentiviral vectors (12 μg) in combination with packaging plasmids pCMV delta R8.2 (12 μg, gift from D. Trono, Addgene #12263) and pHIT/G (6 μg, gift from M.H. Malim, School of Immunology & Microbial Sciences, King’s College, London, Great Britain) via standard calcium phosphate transfection. Thirty-six hours post transfection, lentiviral-supernatants were collected and filtered (0.45 μm pore size) for subsequent infection of DiFi or BoC20 cells in the presence of 4 μg/ml polybrene.

### BEAMing

Purified DNA of PDX mouse model tumors was sent to Sysmex Inostics, Hamburg, Germany. DNA content was quantified using a modified version of LINE-1 Real-time PCR as previously described [48, 49]. An amount of 330 ng DNA per sample was inputted for each tissue BEAMing analysis. BEAMing digital PCR is a technique in which individual DNA molecules are attached to magnetic beads in water-in-oil emulsions and then subjected to compartmentalized (digital) PCR amplification [50]. The mutational status of DNA bound to beads was then determined by hybridization of fluorescently labeled allele-specific probes for mutant or wildtype KRAS G12V (g35t) sequences. Finally, the bead population was analyzed by flow cytometry to count and sort wildtype and mutant beads. The result is reported as the fractional abundance of mutant DNA alleles relative to wildtype DNA alleles per sample. To generate the ratio of mutant to wildtype DNA alleles (mutant allelic fraction, MAF), an average of 3 × 10^6^ beads were interrogated in each BEAMing analysis (approximately 90,000 beads per mutation). The cut-off for calling a mutation in xenograft derived DNA with BEAMing was set to 0.02% MAF.

### Droplet digital PCR

All ddPCR assays were carried out in duplicate according to the manufacturer’s instructions (BioRad Laboratories Inc). For each reaction, a standard volume of 20 μl was prepared using 6 μl DNA containing 50–100 ng DNA and 14 μl mastermix (containing 10 μl 2× Supermix, 1 μl mutation wildtype multiplex assay mixture, 2.75 μl DNA-free water, 0.25 μl (40 U) HaeI restriction enzyme). KR2-12/13 and KR3-61 multiplex screening assays were proved by titration of standard samples purchased from Horizon discovery (H701, Wien; data not shown). The ddPCR amplifications were performed with the following protocol in the BioRad cycler: initial denaturation 95 °C 10 min, 45 cycles with 94 °C 30 s, 55 °C for 1 min, with a ramp rate of 2 °C per sec, and 10 min 98 °C final step. The fluorescence of each droplet was analyzed by QX200 droplet reader, and the data were calculated by QuantaSoft software (BioRad) with Poisson’s algorithm. The assay information of primer sequence context is given at the homepage of BioRad systems.

### Real-time cell analyses (RTCA)

RTCA was performed using 16-well E-plates on the Dual Plate xCELLigence instrument (ACEA Biosciences Inc. San Diego, CA, USA, RTCA software v2.0). This system measures a dimensionless parameter called cell index (CI), which evaluates the ionic environment at an electrode/solution interface and integrates information on cell number. The assays were performed according to the manufacturer’s instructions. Briefly, 100 μl of culture media was added to each well, incubated at room temperature for 30 min and the background impedance was measured. Prior to the RTCA experiments, cells were cultivated in low-serum-medium containing 0.5% FCS for 24 h. For RTCA analyses, cells were seeded at a density of between 5000 and 30,000 cells per well in a final volume of 200 μL into E-plates (16 wells, ACEA Biosciences) and allowed to settle for 30 min at RT before the plates were inserted into the xCELLigence instrument. RTCA assays were performed in a humidified incubator at 37 °C with 5% CO_2_. The growth was monitored at 15-min time intervals for up to 165 h. All measurements were performed in duplicates. After 24 h, selected murine growth factors and/or CET were added. Substances used were cetuximab (Merck KGaA), Fgf9 & 10, Igf2, Pdgfc (RND Systems), Fgf18 (NOVUS Biologicals), Pdgfb, and Hbegf (Sigma-Aldrich).

### Quantitative real-time PCR (qRT-PCR)

cDNA was synthesized using 2 μg of total RNA, oligo(dT)18 primers, and M-MLV reverse transcriptase (Promega) following the manufacturer’s protocol and diluted to a final volume of 50 μl with 1× first strand buffer. Intron spanning primer sets for qRT-PCR were designed using Primer Express 2.0 software (Applied Biosystems) (PPP2R5A-1141-s: AGGATGAACCCACGCTTGAG, PPP2R5A-1233-as: TAGGCTGG AAATCAGGGCTCT, FGF3-693-s: CTGGAGAACAGCGCCTACAGT, FGF3-815-as: CGAAGC ATAGAGTCGTCCCCT). qRT-PCR was performed using a SYBR Green I reaction mixture containing 75 mM Tris-HCl (pH 8.8), 20 mM ammonium sulfate, 0.01 % (v/v) Tween 20, 2 mM magnesium chloride (all Sigma-Aldrich), 1 μl of a 600-fold dilution of SYBR Green I (BioWhittaker), 2.5 U Taq polymerase (NEB), 0.2 mM dNTP (Promega), and 0.2 μM of forward and reverse primer in a final reaction volume of 20 μl. Reactions were run on a CFX Connect Real Time System (BioRad). The cycling conditions consisted of 3 min initial denaturation at 94 °C and 40 cycles of 94 °C for 30 s, 60 °C for 30 s, 72 °C for 30 s, and 80 °C for 5 s. Fluorescence was measured at the last step of each cycle. Melting curves were obtained after each PCR run and showed single PCR products. cDNAs were run in triplicate, non-RT (without reverse transcriptase), and no-template controls were run in duplicates. Expression ratios were calculated as described by M. Pfaffl [51] using the geometric mean expression of the housekeeping gene COX6C (COX6C-108-s: CAGGCGTCTGCGAAATCATA; COX6C-265-as: CAGCCTTCCTCATCTCCTCA GAPDH-s: TGCACCACCAACTGCTTAGC, GAPDH-as: GGCATGGACTGTGGTCATGAGA) to normalize the expression data for the gene of interest.

### Protein extraction and immunoblotting

Cryoconserved xenograft tumors were sectioned at 30 μm using a Leica CM1900 cryostat. Between 20 and 30 sections or cell pellets from 1 × 10^7^ cells were placed in 1.5-ml tubes and lysed in 0.5 ml cold RIPA lysis buffer, supplemented with protease and phosphatase inhibitors (Sigma-Aldrich), the lysates were subsequently sonicated and cellular debris was removed by centrifugation. Protein concentrations of the lysates were determined by the Bradford protein assay system (BioRad). Equal amounts of protein (10–50 μg each lane) were separated by SDS-PAGE and transferred to PVDF membranes (Carl Roth). Immunoblots were blocked with 5% BSA in tris-buffered saline and tween-20 (0.1%, v/v) for 1 h at room temperature. The membrane was incubated overnight at 4 °C with primary antibodies. Antibodies were purchased from cell signaling technology: Total-STAT3 (#4904), P-Y705-STAT3 (#4113), P-T202/Y204-ERK1/2 (#9101), P-S9-GSK-3b (#9323), P-S473-AKT (#4060), P-S62-c-Myc (#13748), P-Y1045-EGFR (#2237), P-Y1068-EGFR (#3777); Abcam: anti-PPP2R5A (ab89621). Anti GAPDH (#2118) and Anti-β-actin (#4967S; Cell Signaling Technology) were used as protein loading controls. The membrane was incubated with the corresponding secondary antibody conjugated with horseradish peroxidase (Dianova). Bands were visualized with enhanced chemiluminescence western blot detection system (Thermo Scientific) together with the ChemiDoc imaging system (BioRad). Signal intensities were quantified using Image Lab software (BioRad). Band intensity was normalized to that of GAPDH or β-actin.

### Gene set enrichment analyses (GSEA)

GSEA software [52, 53] was provided by the Broad Institute of the Massachusetts Institute of Technology and Harvard University (http://www.broad.mit.edu/gsea/). The hallmark gene sets (V6.1) were used with default parameters of the GSEA software package; gene set permutation was used. Gene sets with FDR q-val ≤ 0.05 were considered appropriate.

### CRIS classification

Gene expression data were loaded as data matrix in a linear fashion into the CRIS NTP classifier [54] and run under standard parameters using RStudio (version 1.2.1335) and the CRIS NTP classifier program which is part of the CRISclassifier R package (available via https://www.nature.com/articles/ncomms15107#Sec29).

### Statistical analyses

Unless stated otherwise, results are expressed as the means ± SEM. For the identification of differentially expressed genes, only entities where at least 3 out of the total no. of samples had values within the selected cut-off (50th–100th percentile) were further included in the data analysis process. Using the GeneSpring GX software package version 14.9.1, differentially expressed genes were identified via moderated t-test. The p values were adjusted for multiple testing according to Benjamini and Hochberg [false detection rate—FDR] and results were considered statistically significant at adjusted p values below 0.05. Lastly, only mRNAs with a fold change ≥ 1.7 in the microarray analyses were further considered. All other differences were evaluated by the nonparametric Mann-Whitney U test. Statistical computations were performed using Prism software (Graph Pad, La Jolla, CA, USA).

## Results

### Characterization of the in vivo anti-EGFR secondary resistance PDX platform

**Fig. 1:**
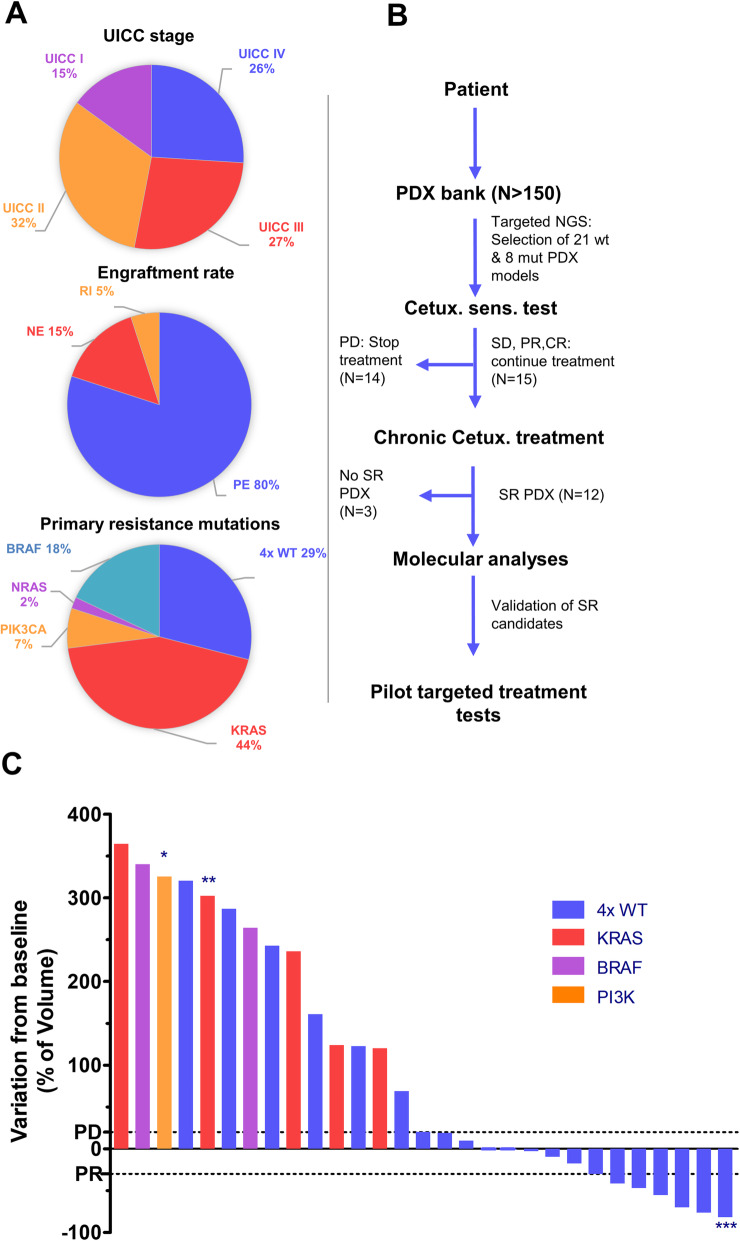
Flow chart and PDX bank characteristics. **a** Engraftment rate of 158 implanted patient tumor samples, frequencies for mutations associated with primary resistance to EGFR treatment from 105 PDX models analyzed by targeted NGS, and distribution of patient tumor stages of the 158 PDX models. PE, positive engraftment, NE, negative engraftment, RI, recently implanted. **b** Scheme for establishing the in vivo anti-EGFR SR PDX models and pre-testing targeted treatment option addressing molecular defined SR mechanisms. PD, progressive disease, SD stable disease, PR, partial response, CR, complete response. **c** Waterfall plot of cetuximab response after 3 weeks of treatment compared with tumor volume at baseline. Dotted lines indicate the cut-off values for progressive disease (PD) and partial response (PR). *,**,***, response at days 7, 15, and 31, respectively

**Fig. 2:**
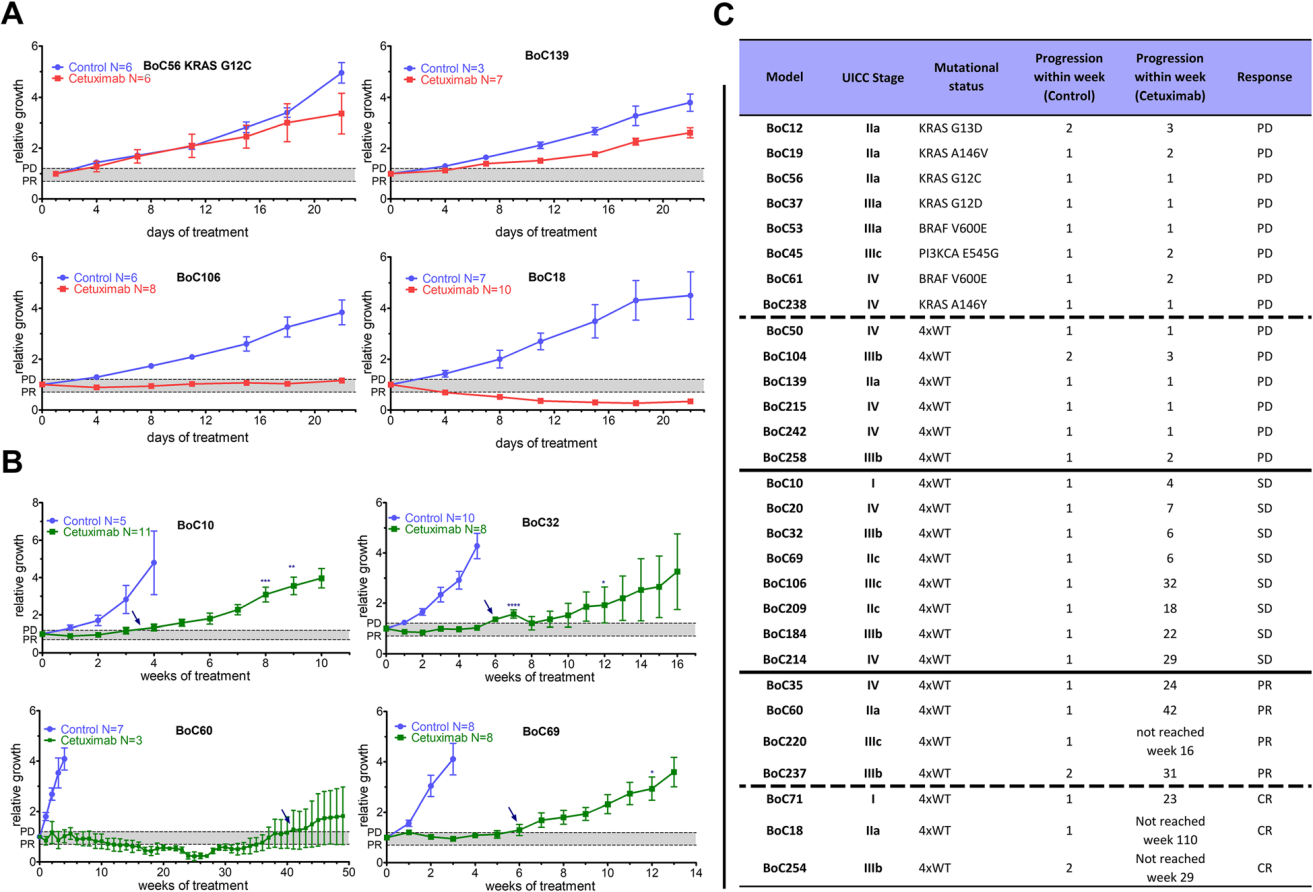
Anti-EGFR SR PDX models can be generated with high efficiency. **a** Representative example of primary responses observed within the initial 21 days of treatment with anti-EGFR mAb (CET) in colon cancer PDX models. PDX model BoC56 with primary resistance due to *KRAS* G12C mutation, PDX BoC139 with primary resistance despite *KRAS*, *NRAS*, *BRAS,* and *PIK3A* wild type status, PDX BoC106 with stable disease, and PDX BoC18 with partial response and quadruple wild type status. **b** Representative growth pattern of PDX models with long-term CET treatment and initial treatment response to anti-EGFR therapy. Time to progression was defined by the mean growth curve crossing the PD borderline. Arrows indicate the time point progressive disease was observed. PD, progressive disease; PR, partial response; gray shades area, stable disease. Relative growth curves are derived from mean values ± SEM (error bars). *, each star represents a tumor that was taken out of the treatment cohort at the indicated time point. Please note that this can lead to marked changes in mean tumor volumes (see also Additional file 4: Table S3 for individual growth curves). **c** Summary of PDX models tested

Since May 2012, 158 patients with CRC undergoing surgery at our centers were prospectively included in our study to establish a representative colorectal cancer PDX cohort (Fig. 1a). We were able to reach an overall engraftment rate of 80%. Passaging of tumors from mice to mice to establish the PDX lines was highly successful (98%) excluding a strong selection bias in our xenograft cohort. Xenografted tumors retained the histopathologic characteristics of original samples (Additional file 2: Fig. S1). We randomly selected 21 PDX models with wildtype sequences based on targeted NGS sequencing in the genes *KRAS*, *NRAS*, *PIK3CA,* and *BRAF* (therefore called quadruple wildtype) as well as 8 PDX models harboring an activating mutation in one of the aforementioned genes to establish their anti-EGFR treatment response characteristics (Fig. 1b, c). In line with previous reports on genetic primary resistance mediators, PDX models with activating mutations in *KRAS, NRAS, PIK3CA,* or *BRAF* did not show any disease control with CET treatment during the primary observational period of 21 days (Fig. 2a, Additional file 2: Fig. S2). Within the quadruple wildtype group, three growth patterns could be observed: progressive disease for 6 PDX models (29%), objective response for 7 PDX models (33%), and stable disease for 8 PDX models (38%) (Figs. 1c and 2a; Additional file 2: Fig. S3-5). The observed primary response pattern frequencies are in line with previous PDX model results and clinical response data [55]. Next, we asked if it is possible to generate in vivo selected secondary resistant tumors in the PDX model via chronic CET treatment for the 15 models (Fig. 1b) showing initial disease control (stable disease and objective response). Indeed, 12 of the 15 models developed secondary resistant tumors defined by disease progression following an initial phase of disease control of at least 3 weeks. The efficiency with which SR PDX tumors evolved in the 12 models varied (Additional file 3: Table S2). In 10 models, we were able to generate two to five independently arising SR, in two models only one SR tumor each, could be established. The main reason for the latter was either health issues of the animals forcing to terminate the experiment (BoC35) or a low number of initial tumors growing in the cohort (BoC60). Similarly, the time to progression in the individual parallel PDX tumors within each model varied by several weeks (Fig. 2b, c; Additional file 2: Fig. S6).

### Genetic drivers of primary and secondary resistance

**Fig. 3:**
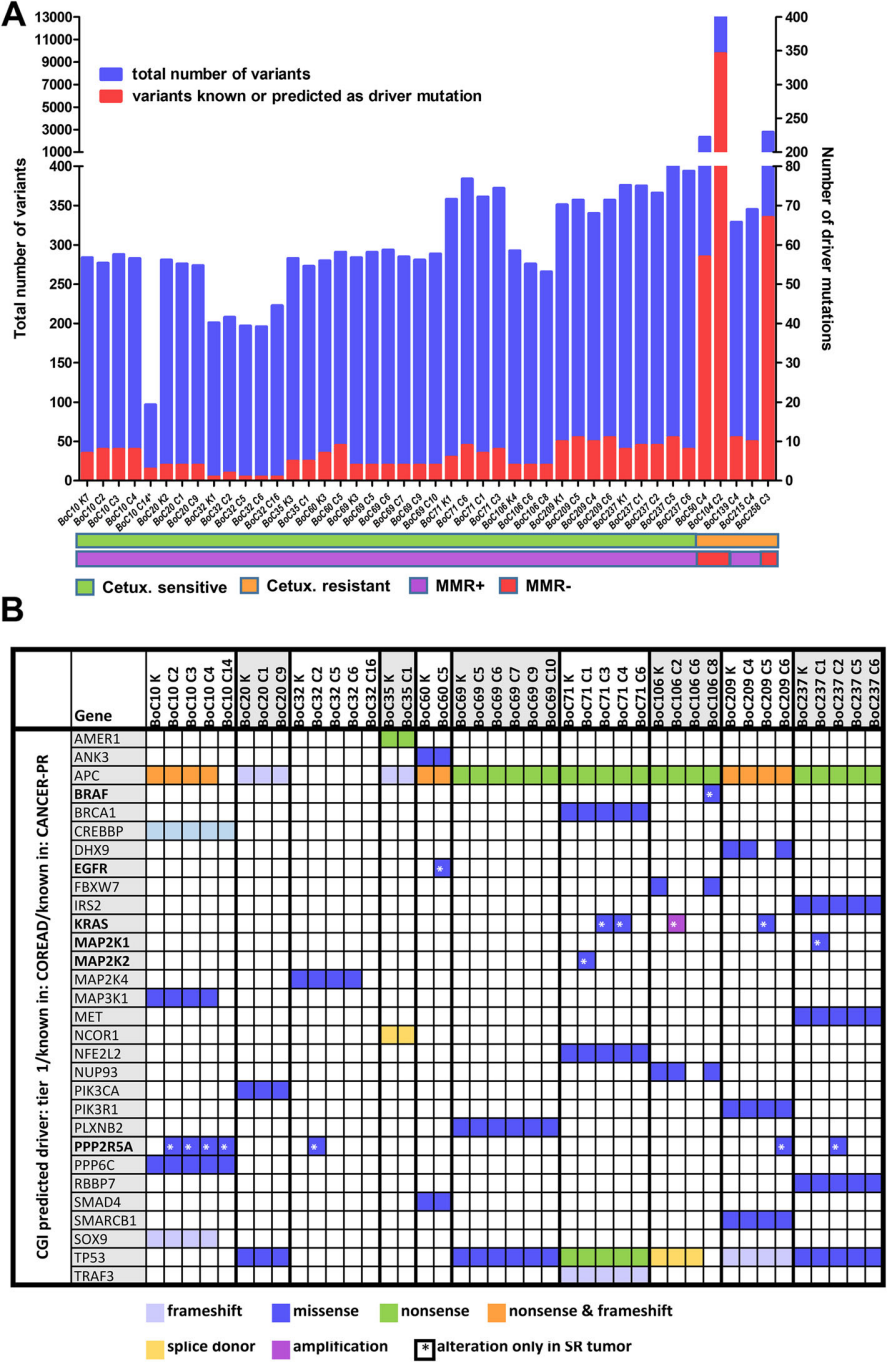
Exome sequencing data of SR PDX models. **a** Non-silent variant load and the thereof derived total number of driver or predicted driver mutations for CET-sensitive and primary resistant models. *, tumor with low sequencing coverage; MMR+, tumors with functional mismatch repair (MMR) system; MMR−, tumors with inactivating mutations in the MMR genes. **b** Cancer gene mutations identified in the 10 PDX models. Acquired mutations in the SR models are indicated by an asterisk. K, untreated control tumor; C, CET SR tumor

**Fig. 4:**
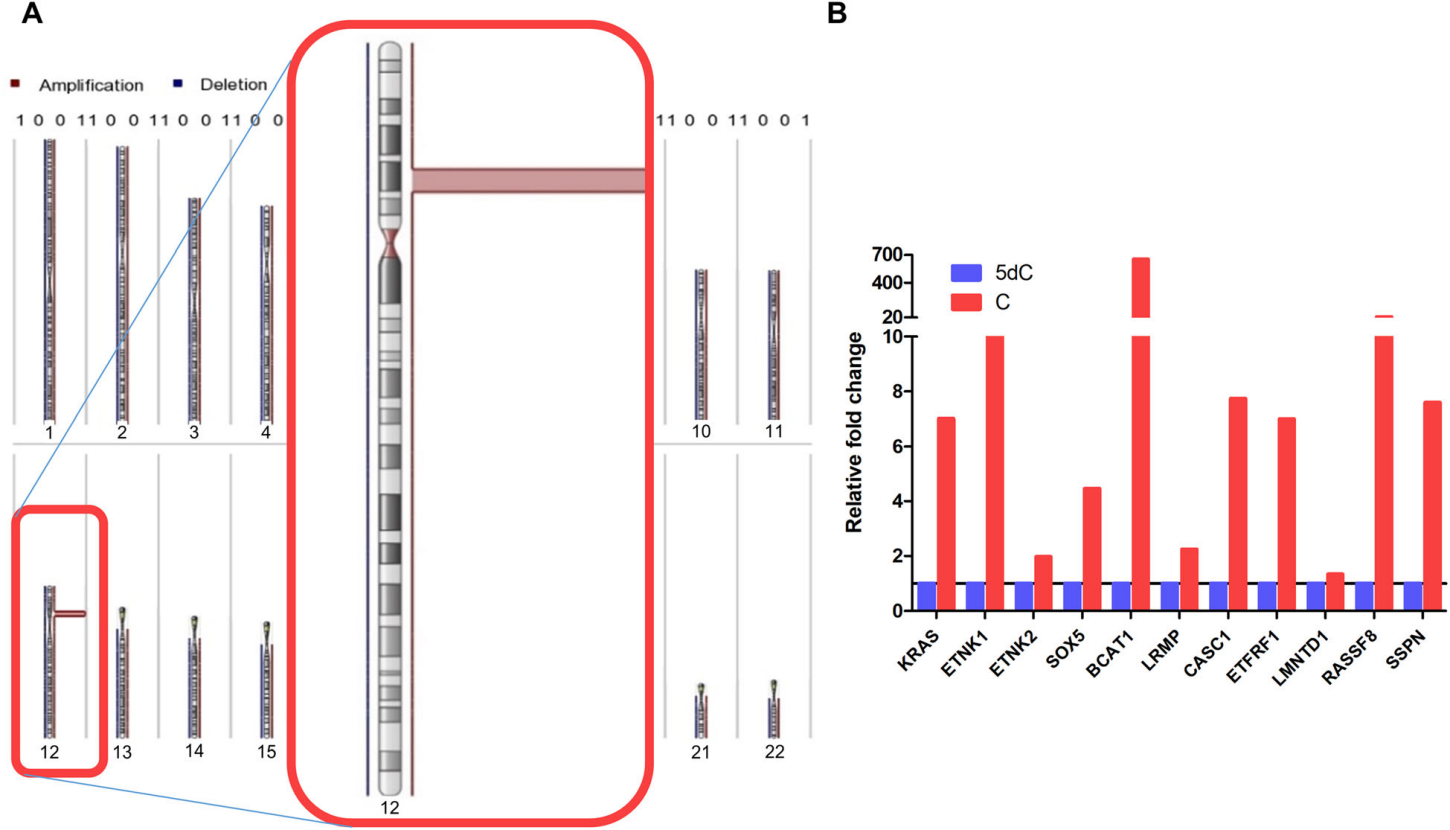
Amplification of chromosome 12p12.1. **a** Schematic view of the amplification targeting chromosome region 12p12.1 (chr12:22615401-26669741) identified in BoC106 C2. **b** Increased gene expression of KRAS and other genes within the amplified region, as measured by array expression profiling. 5dC, tumors treated for 5 days with CET and still sensitive to CET. C, tumors with SR under chronic CET treatment

**Fig. 5:**
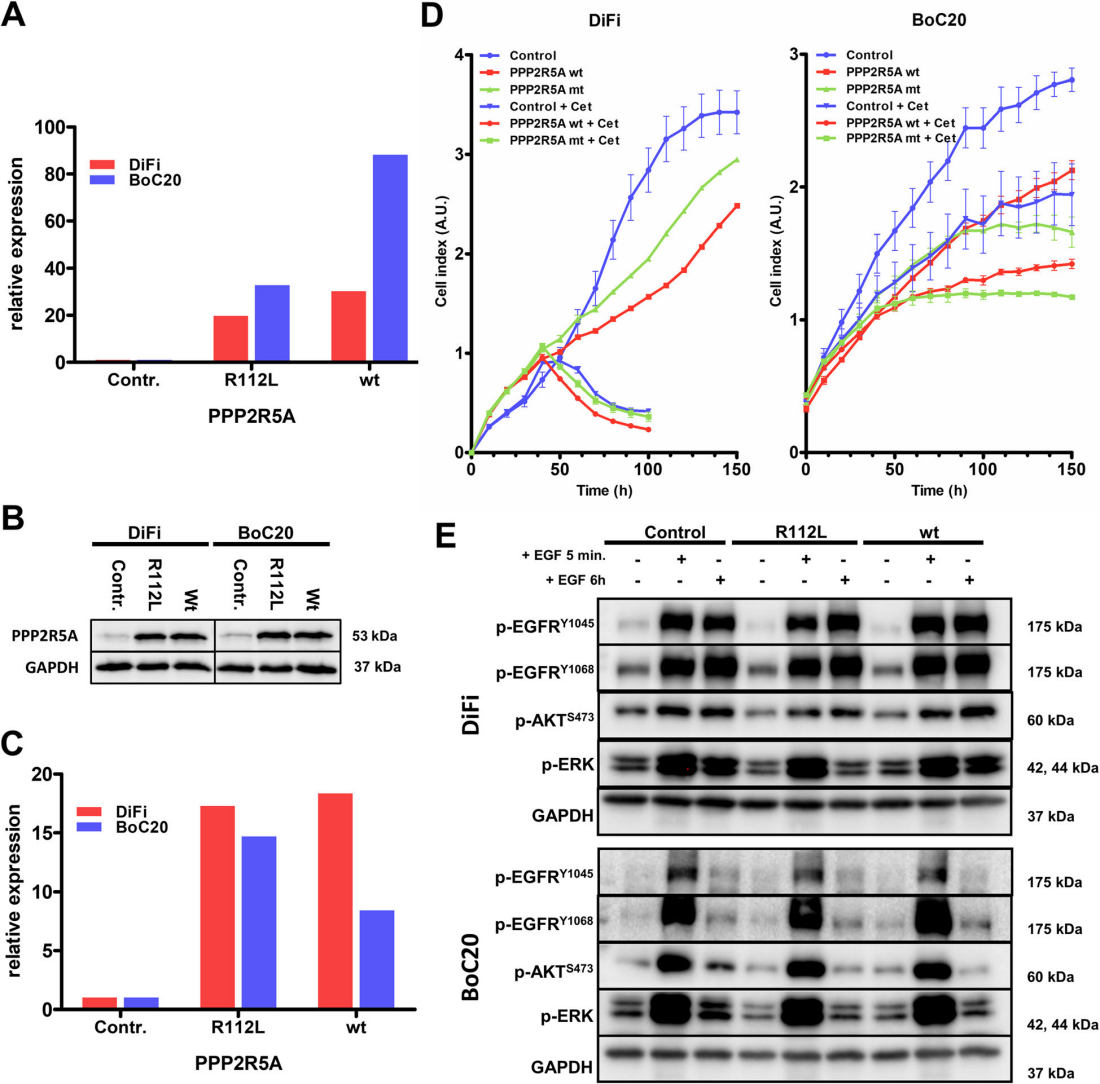
Functional analysis of the PPP2R5A R112L mutation. **a**, **b** qRT-PCR and western blot data confirming the overexpression of either the wt or the R112L mutant *PPP2R5A* transgene. **c** Relative quantification of the indicated protein signal intensities to the corresponding intensities of the control tumor normalized with the corresponding GAPDH signal intensities using Image Lab (BioRad). **d** Proliferation response of colon cancer cells BoC20 and DiFi overexpressing either the wt or the R112L mutant version of *PPP2R5A* with or without CET treatment (DiFi: CET 0.4 μg/ml; BoC20: CET 5 μg/ml). **e** Western blot analysis of pEGFR, pAKT, and pERK following stimulation of serum-starved (0.1% FCS) cells with hEGF (200 ng/ml) for the indicated time. pSFFV-GFP vector transduced cells were used as controls. GAPDH was used as the loading control

First, we re-sequenced untreated control tumors from the first five consecutively arising models with primary resistance via whole-exome sequencing (WES) (Additional file 4: Table S3). The mutational load was similar in all models, except three models with genetic instability due to inactivating mutations in mismatch repair (MMR) genes (Fig. 3a, Additional file 4: Table S3). These MMR-deficient (dMMR) tumors were from the group of primary resistant tumors, with two dMMR models harboring among others inactivating *NF1* mutations paired with either a potentially activating *FGFR1* mutation (K659N, BoC104) or an *HRAS* mutation (R161H, BoC258) and biallelic inactivation of *PTEN* (BoC258). From the two primary resistant and MMR proficient PDX models, BoC215 harbored a mutation in the *ERBB2* gene (S310F), previously described as activating [56]. Taken together, in three of the five primary resistant models, WES revealed potential resistance-conferring mutations, not detected in our initial targeted sequencing approach for selecting quadruple WT tumors. Furthermore, dMMR tumors did not benefit from anti-EGFR therapy. Next, we sequenced all the available 31 SR tumors from the first 10 consecutively arising models either by WES (N = 29) or by targeted NGS (BoC71 C4, BoC106 C2) to identify resistance drivers not present in the corresponding control tumors (Fig. 3b, Additional file 5: Table S4). Furthermore, selected SR tumors from five models (BoC10 C3, BoC20 C9, BoC35 C1, BoC60 C5, BoC69 C5, and BoC106 C2) were analyzed for copy number variations (CNV). In one model (BoC71) with four SR tumors, we found two acquired *KRAS* mutations (G12V & G12C in BoC71 C3 & C4, respectively) and one *MAP2K2* (Q60P) mutation (BoC71C1), while the fourth tumor (BoC71 C6) did not reveal any alterations known to drive SR. A similar genetic heterogeneity in SR conferring driver mutations was found in model BoC106, with one SR tumor (BoC106 C8) carrying a *BRAF* mutation (N518I, described as low-activity or kinase-dead BRAF mutant [57], and therefore not considered as a driver mutation herein), a second (BoC106 C2) developing an amplification (chr12:22615401-26669741) including the *KRAS* gene locus (Fig. 4a, b), while the third tumor (BoC106 C6) did not reveal an obvious driver gene alteration. Additional SR driver gene alterations were identified, including *MAP2K1* (K57N; BoC237; 1 out of 4 tumors), *KRAS* (G12V; BoC209; 1 out of 3 tumors), and *EGFR* (S492R; BoC60 C5). We also observed a recurrent mutation in the tumor suppressor protein phosphatase 2 regulatory subunit B'alpha (*PPP2R5A*; R112L) in 4 SR tumor models (BoC10, 32, 209, and 237). This missense mutation in the beta subunit of the protein phosphatase 2A has been described in COSMIC and is classified as inactivating and highly deleterious in the Cancer Genome Interpreter (CGI). However, overexpression of both the wt and the R112L mutated version of *PPP2R5A* (Fig. 5a–c) had a similar negative impact on colon cancer cell growth and did not alter CET treatment sensitivity (Fig. 5d). Furthermore, pAKT and pERK levels remained at lower levels following EGF stimulation (at 6 h) in cells overexpressing the *PPP2R5A* mutation than in control and wt cells (Fig. 5e). This together with the generally low allele frequency (AF) of mutated *PPP2R5A* (< 35%) found herein, does not support that this mutation conveys *PPP2R5A* loss of function and is unlikely relevant for SR development in our tumors. In the remaining five models including 15 SR tumors, no potential driver gene alteration was identified. Additional mutations in genes with a low predicted driver gene status according to the Cancer Genome Interpreter (CGI) (mainly with tier 2 status and an AF below 20%) were identified in some of the SR tumors. They were not considered SR drivers due to the lack of proof of their functional relevance according to the current literature (Additional file 2: Fig. S7, Additional file 5: Table S4).

### Clonal stability of KRAS G12V SR driver mutation independent of treatment pressure

**Fig. 6:**
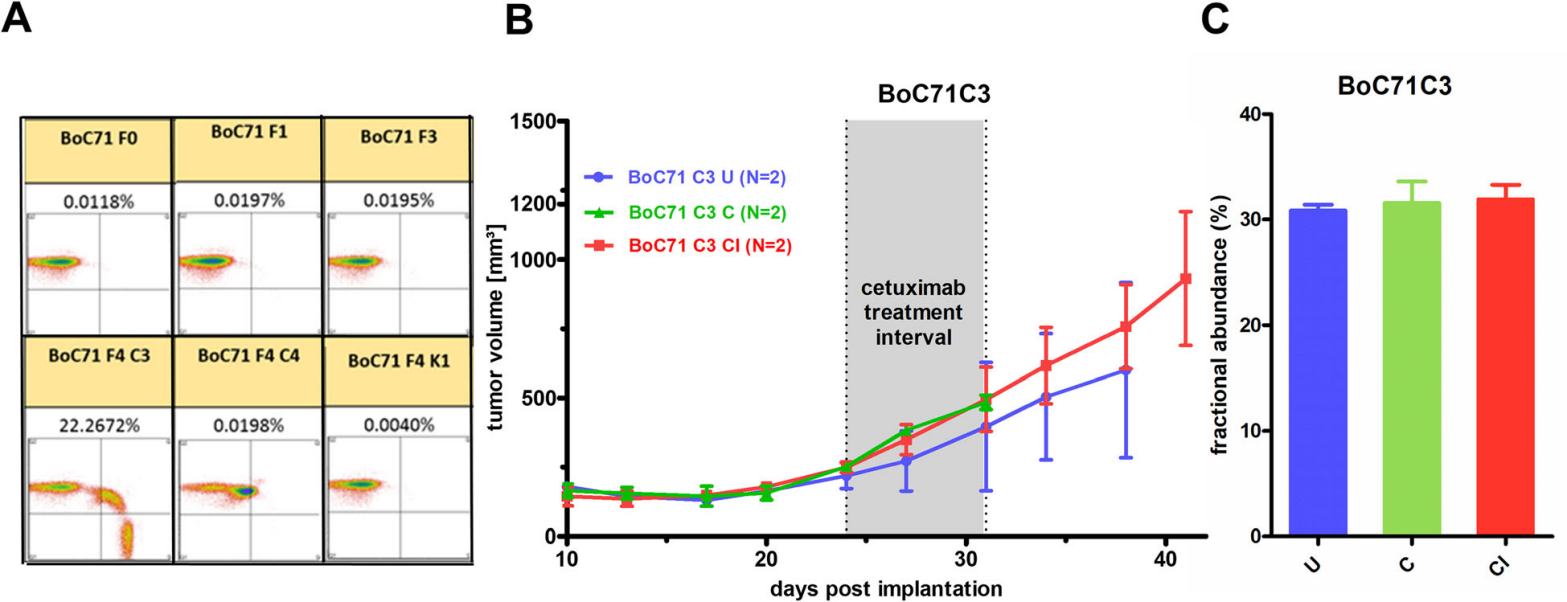
KRAS mutation fraction dynamics under treatment in CET SR BoC71 tumors. **a** *KRAS*-G12V specific BEAMing assay in BoC71 PDX models. Fluorescence dot plots are shown with wildtype population plotted on the Y-axis and mutant population on the x-axis. Only one sample (BoC71 F4 C3) showed a high percentage of G12V mutation, resulting in 22.27% mutant fraction. All other samples are negative for *KRAS* G12V, showing values below our detection limit of 0.02% mutant fraction. Sample BoC71 F4 C4 shows a strong shift of the majority of the wildtype population to the right uncharacteristic for the G12V mutation. This shift stems from the G12C *KRAS* mutation which this single *KRAS* analyte analysis cannot detect. F, PDX generation starting with F0; K, untreated control tumor; 5dC, 5 days CET treated; C, CET secondary resistant. **b** Growth curves of *KRAS* mutated SR tumor BoC71 C3 untreated (U), treated either only for the indicated time frame (shaded gray area) and taken out for analysis at the end of treatment or taken out following a treatment pause and doubling of the tumor volume (CI). **c** Fractional abundance of *KRAS* mutation determined by digital droplet PCR for corresponding experimental set-ups. Data are shown as mean values ± SEM (error bars)

One of the mutated tumors (BoC71 C3) with a KRAS mutation AF of 22 and 26% with BEAMing emulsion digital PCR (Fig. 6a) and WES, respectively, was selected to search for pre-existing *KRAS* G12V mutation by BEAMing analysis up to the 5th generation of treatment-naive BoC71 tumors. Even when using an analytical cut-off of 0.02%, no mutation was detected (Fig. 6a). This data is compatible with both de novo *KRAS* mutation developing under treatment pressure or a very rare pre-existing mutated cell clone below the detection limit of BEAMing as a source of the *KRAS* mutated cells in this anti-EGFR-treated tumor. Previously, ctDNA analyses suggested that *KRAS* driver mutations selected during anti-EGFR resistance development have a fitness disadvantage, as their frequency declines in ctDNA, following a hiatus from anti-EGFR treatment [17, 58]. BoC71 C3 tumor was chosen to test if a similar treatment-dependent alteration in the mutated KRAS allele frequency could be observed. We monitored the mutated *KRAS* AF with high sensitive droplet digital PCR (ddPCR) under treatment and treatment hiatus in BoC71 C3 following re-implantation. First, there was a striking stability of the mutated *KRAS* AF of around 33% between the initially developed SR tumor C3 and all subsequent tumors generated via re-implantation (note that the AF of around 33% in the ddPCR analysis is somewhat higher than in BEAMing and WES, suggesting an overestimation of the AF in the ddPCR for technical reasons). Second, a regimen where initial treatment was followed by a treatment pause and continued tumor growth had no influence on the AF of mutated *KRAS* (Fig. 6b, c). These data support a high stability of the proportion of wt to mutated cells.

### Transcriptional reprogramming during SR development

**Fig. 7:**
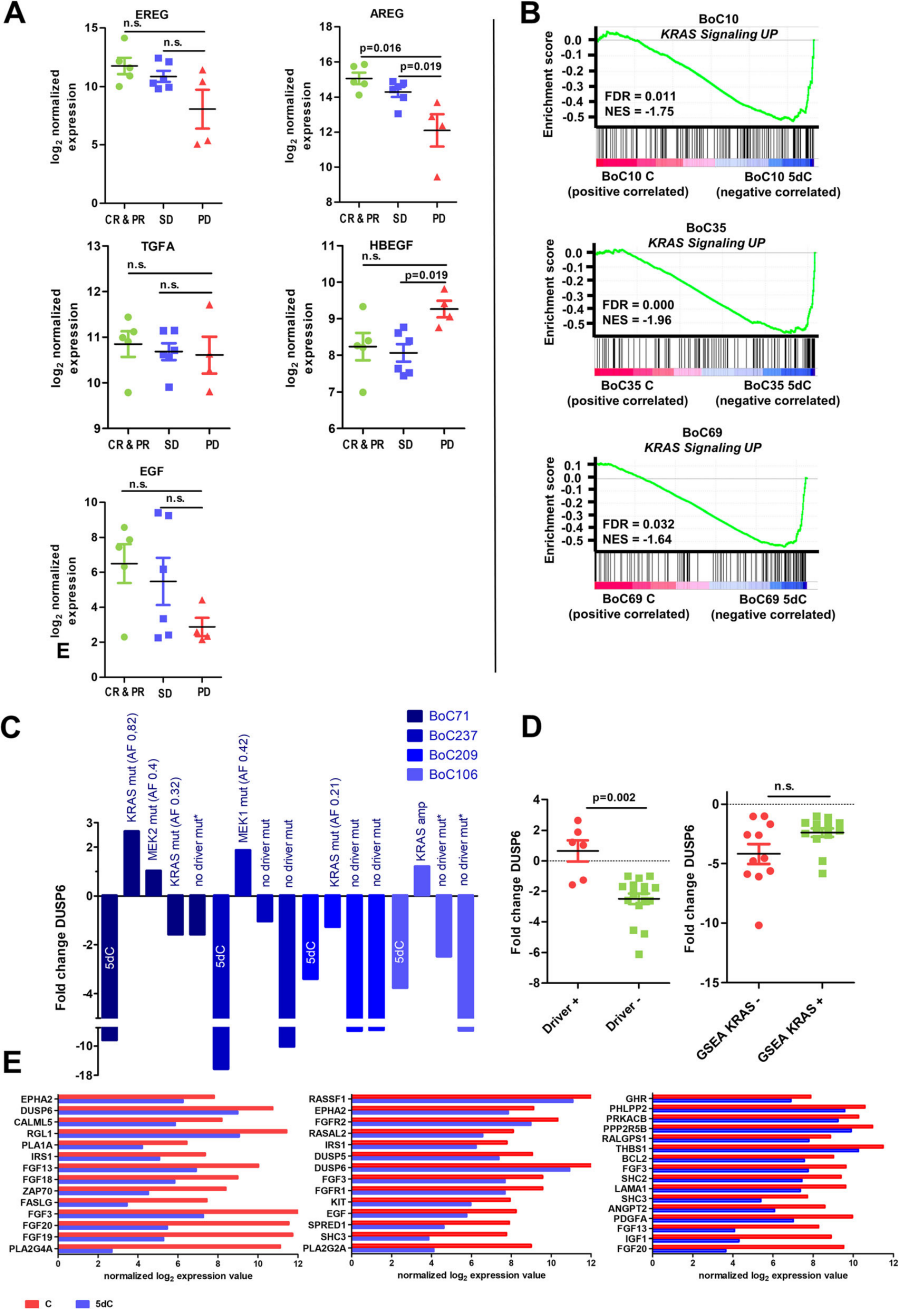
Transcriptomic analyses of SR PDX models. **a** Expression of EGFR ligands in untreated control tumors of the different response groups. CR, complete response, PR, partial response, SD, stable disease, PD, progressive disease. **b** Enrichment plots from GSEA for CET-sensitive compared to resistant PDX tumors showing an enrichment of the hallmark KRAS signaling-up gene set. **c** DUSP6 expression fold change relative to untreated control tumors in SR PDX models with and without driver gene mutations compared to the corresponding CET-sensitive tumors treated for 5 days (5dC). Fold changes were calculated as mean values from at least three measurements. The line indicates the mean. *, models with KRAS_SIGNALLING_UP” set found to be enriched. **d** DUSP6 expression in PDX models correlated with driver mutation and KRAS gene set enrichment status. **e** Upregulation of genes involved in the PI3K-AKT and/or RAS pathway, selected from KEGG pathways. The line indicates the mean. Mann-Whitney U was used to determine significance. 5dC, tumors treated for 5 days with CET and still sensitive to CET. C, tumors with SR under chronic CET treatment

The unexpected low prevalence of SR conferring driver mutations in our set of tumors (23%, 7/31), let us ask whether transcriptional changes may play a role in driving SR in some of the tumors. Therefore, global gene expression profiles of all 31 tumors with acquired SR were established via standard array technology. Comparing the profiles of SR tumors with the corresponding profiles of the CET-sensitive control tumors, collected at day 5 of treatment, identified numerous transcriptional changes in the SR tumors (Additional file 3: Table S2). First, we classified sensitive tumors as well as their corresponding SR tumor according to CRIS classification [54], a categorization system optimized for xenograft tumors (Additional file 2: Fig. S8). As expected, none of the untreated control tumors fell into CRIS-class A, which was associated with molecular alteration such as *BRAF* or *KRAS* mutations [54]. Class switches between the CET-sensitive short-term treated tumors (5dC) and the corresponding SR tumors were with the exception of four SR tumors not prevalent. Furthermore, AREG and EREG, but not the other three known EGFR ligands, were shown to be positive modulators of primary anti-EGFR response and treatment outcome, both in patients and CRC PDX models [59–61]. These EGFR ligands are considered to induce a stronger EGFR dependency in colon tumors which renders them particularly sensitive to anti-EGFR treatment. Accordingly, AREG was expressed at significantly higher levels in control tumors of CET responders versus tumors of non-responders (Fig. 7a). A similar correlation could not be demonstrated for the remaining EGFR ligands. We also searched for overexpression of genes of the ERBB receptor family, the MET receptor, the RAS-RAF-MEK-ERK or the PI3K-AKT pathway, some of them were previously shown to be amplified in anti-EGFR SR tumors. In line with the demonstrated Chr. 12p12.1 amplification, BoC106 C2 showed a strong increase in KRAS expression (Fig. 4b). However, there was no indication of an amplification of the aforementioned genes in any of the remaining SR tumors, because gene expression levels did not vary by more than 2-fold between untreated controls (K) and resistant tumors (Additional file 2: Fig. S8). We also noted three PDX models (BoC10, 69 and 209, Additional file 2: Fig. S8) with a high basal IGF2 expression. In line with previous reports, all three models belonged to the SD response group [61].

### Gene set enrichment analyses to uncover resistance pathways

Having excluded known mechanisms of resistance for the majority of our SR tumors, we next searched for classes of genes that are over-represented in the transcriptome of SR tumors and which may, therefore, have an association with the resistance phenotype via Gene Set Enrichment Analyses (GSEA) for “hallmarks of cancer.” In agreement with previous reports, showing that reactivation of the RAS pathway is key to anti-EGFR SR development, the “KRAS_SIGNALLING_UP” set was found to be enriched in SR tumors without a genetic driver alteration in five of ten tumor models (Fig. 7b; Additional file 3: Tables S2, Additional file 6: Table S5). Dual specificity phosphatase (*DUSP6*) is central in the negative feedback regulation of the KRAS signaling pathway and its expression level was therefore used as a surrogate marker gene for RAS pathway activity. In line with RAS signaling attenuation, we observed in all 10 models a 2.2- to 16-fold reduced DUSP6 expression during the initial CET treatment phase (5dC) as compared to baseline (untreated) tumors (Fig. 7c; Additional file 2: Fig. S9). Upon SR development, DUSP6 was significantly higher expressed in tumors with driver mutations (Fig. 7d). Importantly, DUSP6 expression levels rose above baseline expression in tumors with near clonal to clonal selected driver mutations (here AF ≥ 0.4) or amplifications, but remained below baseline in tumors with an AF ≤ 0.3 (Fig. 7c). Furthermore, tumors with enrichment of the “KRAS_SIGNALLING_UP” set were more likely to have a higher DUSP6 expression as compared to the group of tumors not showing this enrichment, corroborating the GSEA data (Fig. 7d). Among the identified and confirmed upregulated genes that are able to activate the KRAS signaling pathway, growth factors (GFs) and their receptors initiating an autocrine growth loop via *FGF3, 9, 18, 19,* and *20*; *FGFR1* and *FGFR2*; *IGF1, IGF2, KIT*, and *PDGFA* are according to our data likely playing a prominent role in acquired SR (Fig. 7e; Additional file 2: Fig. S8, S10, and S11, Additional file 3: Table S2, Additional file 7: Table S6). Further, differentially regulated candidate genes in the RAS pathway were the ras guanine nucleotide exchange factor (GEF) *RASGRF1*, the calcium-dependent phospholipid-binding protein *PLA2G4A,* and the downregulation of the RAS-GTPase activating protein *RASAL1* (Fig. 7e; Additional file 2: Fig. S10, Additional file 3: Table S2, Additional file 7: Table S6).

Additional, potentially cooperating pathway alterations in five of the ten resistant tumors (Boc10, 60, 69, 106, and 237) could provide TGF-beta signaling inhibition via the WNT target gene *BAMBI* and/or the *IRX3* and *IRX5* genes (Additional file 2: Fig. S10, Additional file 7: Table S6). These genes have been shown to play a role in colon cancer development and may thus provide the cell with some growth advantage under anti-EGFR therapy, supporting resistance development [62–64].

A subgroup of three resistant tumors (BoC20, 60, and 69) showed markedly higher expressed genes involved in cancer stem cell signaling, such as *OLFM4, CXCR4, ALDH1A1,* or *ALDH1A3*. This was accompanied by a striking upregulation of defensins (*DEFA5* and *DEFA6*) together with an activation of the Notch pathway via the ligand *DLL1* in two of the three tumors (Additional file 2: Fig. S10, Additional file 7: Table S6). This gene expression pattern shows similarities to what has been described for colonic paneth-like secretory cells. These cells are known to play a role in stem cell maintenance and may thereby support resistance towards anti-EGFR antibodies [65, 66]. Interestingly, a strong reduction of *HOXA5* gene expression was also observed in BoC60, a gene recently shown to be involved in intestinal stem cell fate control [67].

### Paracrine support of SR from the mouse stroma

**Fig. 8:**
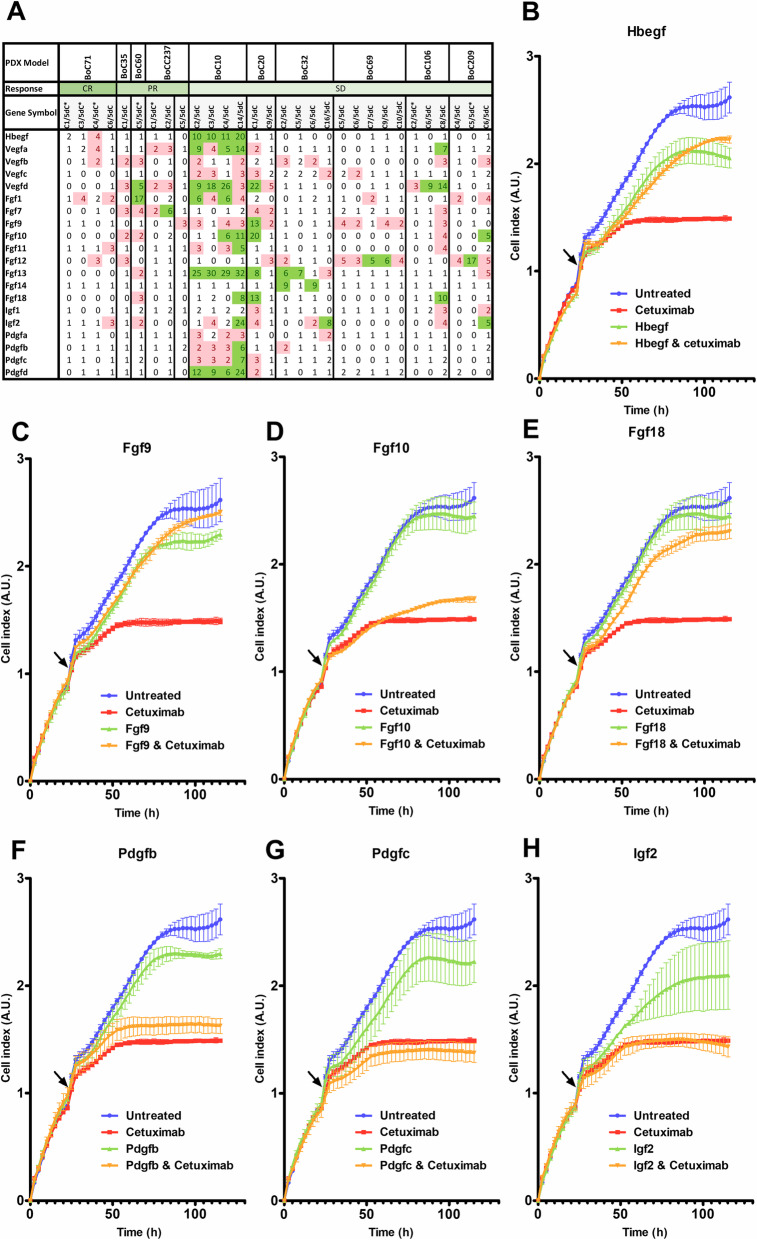
Mouse growth factors impair CET response. **a** Fold changes for selected GFs showing an increase in expression in SR tumors (C) relative to their corresponding tumors after 5 days of CET treatment (5dC). *, SR tumors with driver gene alterations. CR, complete response, PR, partial response, SD, stable disease. **b–h** Proliferation response of primary colon cancer cells BoC20 upon treatment with the indicated GFs (Fgf9: 19 ng/ml; Fgf10: 240 ng/ml, Fgf18: 100 ng/ml, Pdgfb: 10 ng/ml; Pdgfc: 700 ng/ml, Igf2: 10 ng/ml; Hbegf: 10 ng/ml) and CET (5μg/ml) alone or in combination measured via RTCA. GFs and/or CET were added at the indicated time point (arrow). All cell index measurements were taken in duplicates and are shown as mean values ± SD (error bars). A.U., arbitrary unit

It is well established that mouse PDX models maintain the architecture of the original tumor and replace human stroma with mouse stroma within the first two generations of PDX [68], supporting that human epithelial and mouse stroma cells establish a functional communication. Therefore, we used high coverage 3’-RNA-seq to address whether, i.e., GF expression is altered in the stroma derived from SR tumors as compared to control tumors at day 5 of treatment. Indeed, a rise in GF expression, mainly Fgf- but also Egf-, Igf-, and Pdgf-family members, was frequently observed (Fig. 8a), suggesting an additive paracrine activity of the stroma cells towards SR development, at least in a subset of tumors. We used real-time cell analyses (RTCA) to test several recombinant mouse GFs (Fgf9, 10, and 18, Pdgfb and c, Igf2, Hbegf) for their ability to reduce CET response in a quadruple wt primary cell line derived from BoC20. Indeed, mouse GFs Fgf9, 18, and Hbegf strongly interfered with the growth-inhibiting activity of CET (Fig. 8b–h), with Fgf9 and 18 showing the highest homology at the amino acid level (> 98%) with the corresponding human GF among the tested GFs.

### Baseline GF expression levels are linked to anti-EGFR Response

**Fig. 9:**
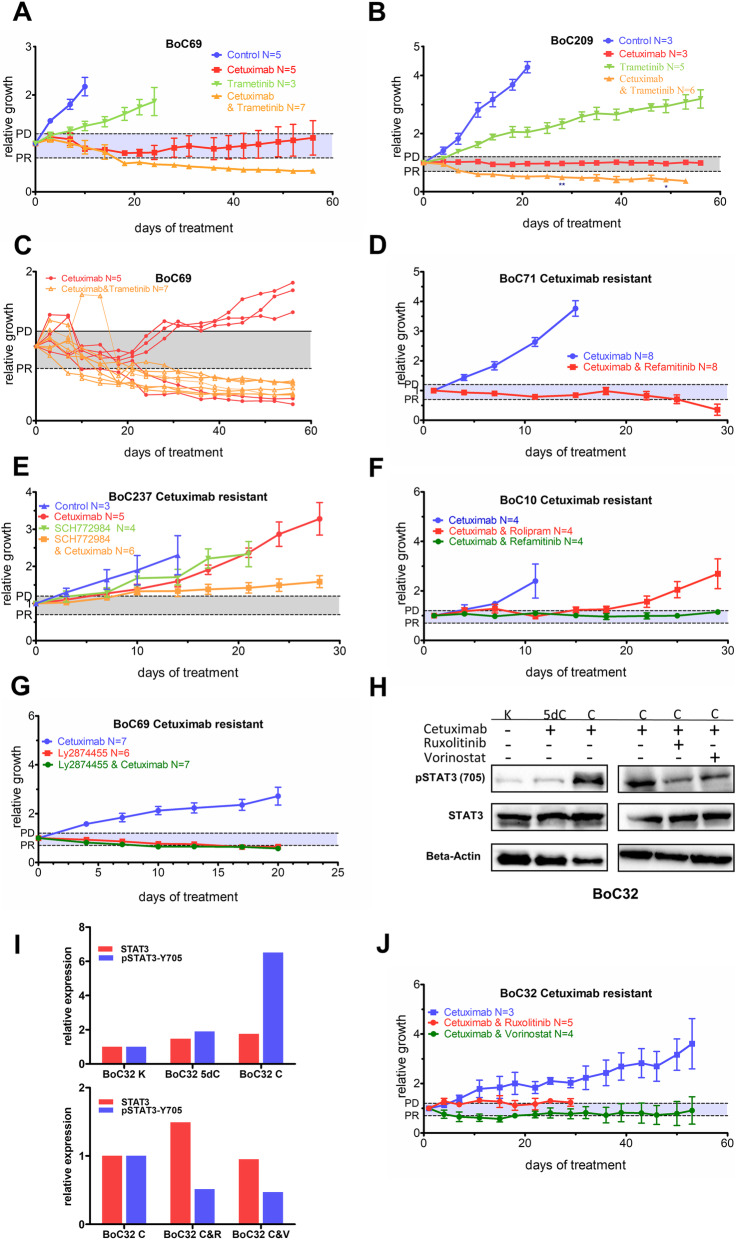
In vivo targeted treatment tests. **a, b** CET-sensitive models with increased baseline GF expression were treated with the indicated mono- or combination therapy aiming at response optimization and delay of SR development. *, each star represents a tumor that was taken out due to weight loss of the mouse. **c** Individual growth curves for tumors under either CET mono- or CET-trametinib combination therapy from panel **a**. **d–g** The indicated CET SR models were treated with the indicated mono- or combination therapy addressing identified SR mechanisms. PD, progressive disease; PR partial response; gray shaded area, stable disease. **h** Representative immunoblots of total STAT3 protein expression and phosphorylation of STAT3 at Y705 [pSTAT3 (Y705)]. K, untreated BoC32 tumors; 5dC, tumors treated for 5 days with CET; C, tumors which developed SR under chronic CET treatment (left panel) as well SR tumors treated with the indicated mono- or combination therapy (right panel). **i** Relative quantification of the indicated protein signal intensities to the corresponding intensities of the control tumor normalized with the corresponding beta-actin signal intensities using Image Lab (BioRad). Relative growth curves are derived from mean values ± SEM (error bars); V, Vorinostat; R, Ruxolitinib. **j** The BoC32 CET SR model with high pSTAT3 was treated with the indicated mono- or combination therapy addressing activated STAT3 signaling

We noted that three treatment-naive PDX models (BoC10, 69, and 209) exhibited in addition to the expression of EGFR ligands a high expression of IGF2 as well as several FGFs, and PDGFs GF family members not observed in the other models (GF^high^) (Additional file 2: Fig. S8). Furthermore, all three models responded to CET treatment with stable disease. We hypothesized that the additional GF signaling is, among others, likely increasing the RAS pathway activity, not targeted by CET. Therefore, we tested for two models (BoC69 and 209) whether the addition of a MEK inhibitor would deepen the response and furthermore delay SR development. Indeed, we observed, that both PDX models could be shifted in the PR response group via this combination (Fig. 9a, b). Importantly, while three of five BoC69 tumors under chronic CET monotherapy gave rise to SR tumors, none of the seven tumors developed SR under the combination therapy (Fig. 9c). Of note, while prolonged trametinib monotherapy was well tolerated, its combination with CET led to an increase in toxicity with two of eight mice experiencing weight loss forcing us to terminate the experiment while in the remaining 6 mice body weight remained stable throughout the therapy (Additional file 2: Fig. S12). This underpins that careful dose-finding is required to balance therapeutic efficiency with toxicity for this combination.

### Functional proof of selected secondary resistance mechanisms

In BoC71 C3, we addressed the *KRAS* G12V mutation, a well-known secondary resistance mechanism. This model was therefore treated with the combination of an anti-EGFR antibody (CET) and a MEK inhibitor (refametinib) to achieve optimal vertical inhibition. In line with previous publications, BoC71 C3 reached partial response after 4 weeks of treatment, proving the efficacy of MEK-EGFR co-targeting (Fig. 9d) [10, 69–71]. In BoC237, the identified MAP2K1 C171G mutation was addressed with the highly selective and ATP-competitive ERK1/2 inhibitor SCH772984. In contrast to previous reports in a BRAF mutated cell line [72], ERK-I monotherapy failed to control tumor growth at a dosing of 12.5 mg/kg bid whereas the combination of CET with SCH772984, albeit it did not reach our criteria for stable disease, somewhat controlled tumor growth, but was unable to induce tumor shrinkage (Fig. 9e). Doubling of the SCH772984 dose in combination with CET was poorly tolerated, and an initiated treatment trial was discontinued.

To show that the identified transcriptional changes are indeed conferring SR, we chose to therapeutically address in a proof-of-concept experiment two novel anti-EGFR secondary resistance mechanisms, which to date have not been described for CRC in this context, involving the cAMP and FGFR signaling pathways. Secondary resistant tumor BoC10 showed a strong upregulation of two family members of cAMP-specific phosphodiesterases (*PDE4B* and *PDE4D*) of 206 and 15-fold, respectively, compared to the 5-day controls. The phosphodiesterase family is known to degrade the secondary messenger adenosine and guanosine 3′,5′-cyclic monophosphates (cAMP and cGMP, respectively). PDE4D may support proliferation in prostate and lung cancer [73, 74], and cAMP is able to augment or suppress ERK activity in a cell type-dependent manner [75]. This secondary resistant tumor was therefore treated with a combination treatment of CET and rolipram, an unselective PDE4 inhibitor. This treatment controlled the growth of this SR tumor for 2 weeks (Fig. 9f) supporting a previously undescribed role of phosphodiesterases in SR development for a subgroup of tumors. Since this combination was unable to sustain tumor growth control over a longer period of time (Fig. 9f), we searched for alternative treatment options. In this tumor, we also observed not only upregulation of FGF ligands (FGF19 and FGF20; 88- and 68-fold, respectively) but also a very strong upregulation of the phospholipase *PLA2G4A* (345-fold). These genes are able to activate among others the MEK-ERK signaling pathway [76, 77]. We therefore also tested the combination of CET and refametinib. We achieved disease control until treatment was stopped after 28 days (Fig. 9f). This indicates in agreement with the GSEA data that vertical inhibition via blocking KRAS signaling in combination with controlling the feedback activation of the EGFR receptor phosphorylation via CET was critical to control this tumor. Another tumor model BoC69 developed a remarkably high expression of *FGF20* and *FGF13* (59- and 18-fold, respectively). As an autocrine GF loop driven by FGF appeared to be the SR mechanism most likely in this tumor, we chose to test FGFR signaling blockade via the pan-FGFR inhibitor (FGFR-I) LY2874455. FGFR-I monotherapy achieved partial response which was surprisingly good and this was not improved by adding CET, suggesting a switch in GF dependencies for tumor growth from EGFR to FGFR signaling in this tumor (Fig. 9g).

In the tumor model BoC32, we observed in SR tumors an upregulation of IL-6 family members such as Ciliary Neurotrophic Factor Receptor (*CNTFR*) and Interleukin 6 receptor (*IL6R*) (Additional file 7: Table S6), known to be able to signal via Janus kinase (JAK)/(STAT) [78]. Therefore, we performed in addition phosphoprotein Western blot analyses for STAT3. We found a strong increase in the STAT3 phosphorylation signal in the secondary resistant tumor in three of the four SR PDX tumors generated (Fig. 9h, i; Additional file 2: Fig. S13). Since clinically usable STAT3 inhibitors are still under development, STAT3 activated resistant tumor BoC32 C2 was treated in combination with CET and ruxolitinib, a JAK1/2 inhibitor or with the histone deacetylase (HDAC)-inhibitor vorinostat both of which have been shown to cause an inhibitory effect on STAT3 signaling [79, 80]. With both substances, we observed disease control, which was more pronounced with vorinostat than with ruxolitinib (Fig. 9j). Vorinostat was therefore tested for an extended treatment period. In line with the expected effect of both drugs, we found a sustained reduction of pSTAT3 following both treatments (Fig. 9h, i). In contrast to previous reports linking high pre-treatment pSTAT3 activity as well as the primary failure of CET to reduce STAT3 transcriptional activity to poor primary anti-EGFR response [81, 82], our data show that rewiring of signaling during chronic CET therapy can induce a high STAT3 activity status likely supporting SR development.

## Discussion

Secondary resistance is the main reason for acquired treatment failure to targeted therapies. Here, we investigated the feasibility to generate in vivo selected CET SR PDX tumors from CRC patients as a tissue source for in-depth molecular analysis of SR mechanisms. We found that the pre-selection of CRC tumors for their *KRAS*, *NRAS*, *BRAF,* and *PI3K* wildtype status efficiently selected anti-EGFR sensitive tumors in the majority of PDX models tested (15/21). Furthermore, SR tumors were established with a high success rate (80%) from CET-sensitive PDX models. This enabled us to generate altogether 31 SR tumors and thus the largest to date available CET SR PDX collection of in vivo selected SR tumors derived from primary patient tumor tissue. WES identified a high mutational load due to MMR gene inactivation only in the CET primary resistant cases, adding a note of caution for treating MMR-deficient tumors with CET. A recent single-center retrospective analysis supports this observation [83]. Importantly, WES detected driver gene alterations only in 23% of 31 CET SR tumors, including acquired *EGFR*, *KRAS, MEK1*, and *MEK2* mutations as well as one case with a *KRAS* amplification. The unexpected low frequency of driver mutations in SR let us ask whether transcriptome analyses may help to explain how driver mutation-negative PDX tumors acquired resistance. We observed a widespread transcriptional reprogramming of tumors during SR development. Using the CRIS classification system, we noted only a few occasions of class switches from sensitive to SR tumors, indicating that a consistent shift in cancer cell-intrinsic transcriptomic subtypes cannot be linked to SR development. In agreement with the concept that RAS pathway activation is critical for anti-EGFR SR development [10], GSEA and pathway analyses supported that in half of our SR tumors the observed transcriptional reprogramming leads to reactivation of the KRAS signaling pathway also accompanied by activation of the negative feedback via DUSP6. Transcriptional reprogramming and selection of tumor cells with an increased *FGF-, IGF*-, and *PDGF-* GF-family member gene expression inducing a signaling bypass to the CET EGFR blockade was identified as likely driver of SR in a subset of SR tumors. Constitutive overexpression of a few GFs such as *IGF1*, *IGF2*, and *FGF9* prior to anti-EGFR therapy has previously been described to modulate the intrinsic resistance towards anti-EGFR therapy [61, 84, 85]. In addition, overexpression of downstream tyrosine kinase receptors including *FGFR1*, *FGFR2, KIT*, and *PDGFRA* was identified as a genetic primary resistance mechanism [13, 86]. Very few studies have to date analyzed pairs of pre-treatment and anti-EGFR SR tumors lacking driver gene mutations at the transcriptome level. One study from Woolston et al. very recently suggested that overexpression of GFs from the FGF- and TGF-β family are likely playing a role in SR development [14]. Importantly, they proposed cancer-associated fibroblasts (CAFs) as the main source for the observed rise in GF expression, supported by the switch from the CMS2 to the CMS4 subtype in SR tumors, a subtype known to be enriched for fibroblasts [87]. Thus, in his model, SR is driven by stromal remodeling and paracrine activity of a GF-rich stroma. The use of PDX models enabled us to differentiate between human and mouse cells as a source of the GFs and therefore differentiate between auto- and paracrine signaling, which is difficult to assess in primary human tumors. In contrast to Woolston et al., our data show for the first time that SR development is supported via transcriptional reprogramming of the tumor cell itself establishing autocrine GF signaling, which is further augmented by GF derived from tumor stroma. We also noted a subset of tumors with a higher baseline epithelial GF expression (GF^high^)—mainly FGF-, IGF-, and PDGF-family ligands—with some tumors developing SR by further increasing the expression of GF belonging to the same GF family and thus activating the RAS-RAF-ERK pathway independent of EGFR signaling. In a first pilot experiment, combined MEK and EGFR inhibition was not only able to improve primary response in GF^high^ tumors but prevented SR development, suggesting that transcriptomic data of pre-treatment primary tumors may help to identify such GF^high^ tumors, which may benefit from this combination therapy. Clearly, a more systematic analysis is needed to fully evaluate the impact of baseline GF expression on CET response and SR development.

In the only additional study available probing SR patient tumors via transcriptomic analyses, one tumor was found with enrichment in epithelial-to-mesenchymal transition (EMT) signature, and in another, a drop in the stromal infiltration signature accompanied by an increase in the immune infiltration signature, while in the third analyzed tumor, no signature enrichment was observed [12]. In line with this data, we also observed enrichment of the EMT signature in a subgroup of our SR models (3/10), only (BoC10, 20 and 106, Additional file 6: Table S5), while Woolston et al. reported an EMT expression signature enrichment in the majority of his SR tumors (5/7). Future transcriptome analyses need to include higher numbers of cases in order to define the respective ratios regarding the transcriptional landscape in anti-EGFR SR and the pathways involved.

Lastly, in a proof-of-concept experiment to evaluate the functionality of identified targets, we selected prominent resistance mechanisms including activation of the cAMP-, FGF-, and STAT3-pathway for in vivo targeting. In all instances, we were able to regain control over tumor growth following targeted inhibition of these pathways, proving that the observed transcriptional reprogramming supports SR development. Of note, in these experiments, PDX tumor growth to reach the required volume for treatment initiation was done without the presence of CET. Nevertheless, in all instances, the anti-EGFR SR phenotype was readily established upon CET treatment, suggesting that the non-genetic resistance mechanisms discovered herein are likely stable and heritable.

Moreover, our strategy to generate in vivo SR models via chronic anti-EGFR treatment was able to successfully mimic the parallel evolution of multiple resistant lesions per patient tumor, represented in our setting by the multiple SR tumors evolving in the majority of our tested PDX models. Furthermore, heterogeneous molecular SR mechanisms among different SR tumors originating from the same primary tumor were also prevalent. This supports that SR PDX tumors are a good approximation to the human situation. Besides, the low frequency of resistance driver mutations found in this study is in good agreement with several analyses in primary CET SR tumor tissues reporting a low to moderate *KRAS* mutation frequency (9–21%) [4, 5, 8, 12–14]. However, it is in marked contrast to the current circulating tumor DNA (ctDNA) literature which suggests that pre-existing or de novo generated *RAS* and de novo *EGFR* mutations are the primary cause of SR development in up to 96% of CRCs [9, 15, 17, 58, 88–93]. The discrepancy between ctDNA and primary tissue mutation data has so far not been resolved. While mutation data from primary tissues are generally derived from one representative tissue biopsy only per patient, ctDNA analyses are considered to be an integrated molecular proxy to the overall patient’s SR lesions. However, ctDNA analyses are currently technically limited to the detection of mutations and in consequence unable to detect non-mutational SR mechanisms, as found herein. Our data support that a good proportion of the resistance driver mutation-negative tissue biopsies are from truly mutation-negative SR lesions and are likely driven by transcriptional reprogramming. Therefore, optimizing treatment by targeting the mutation pattern identified via ctDNA analysis is likely to fail in patients with progressing lesions lacking a resistance driver mutation.

The percentages of mutated *KRAS* alleles in ctDNA have repeatedly been reported to decline when anti-EGFR treatment is withdrawn [9, 17, 58], indicating in these cases a fitness disadvantage of *KRAS* mutated cells. We found a striking stability of the mutated *KRAS* AF after re-implantation of the SR tumor in all treatment schedules tested. Our data do not argue against the existence of such a fitness disadvantage but suggest that it may be relevant in a subgroup of tumors, only. In light of the discrepancy in driver mutation frequencies detected in ctDNA and primary tissue, a more direct confirmation of the decline of driver mutation AFs in SR primary tissues is warranted, not least because anti-EGFR re-treatment strategies have been suggested based on the ctDNA data. Considering that the observed decline of mutated *KRAS* alleles in ctDNA upon CET treatment cessation was not consistently accompanied with tumor shrinkage [58], another intriguing possibility may also in part explain a proportion of the observed discrepancy between ctDNA and primary tissue mutation data. It is well conceivable that driver gene activation, as well as transcriptional reprogramming, can arise in parallel in the same lesion and compete with each other or happily coexist.

The high frequency of SR tumors lacking a resistance mutation identified in our PDX models is compatible both with the presence of proliferating cancer cell subpopulations with a reversible drug-tolerant state and slow proliferating drug-tolerant persister (DTP) cells [94–100]. In the first model, anti-EGFR treatment in CRC would induce stepwise transcriptional reprogramming of pre-resistant cells into a stably resistant state [95]. In the second model, slow cycling DTP cells initially survive anti-EGFR treatment and later start to undergo cell division and acquire de novo resistance mechanisms, including driver and non-driver resistance mechanisms in a subset of persister cells, as recently shown for a number of tumor types [94, 97–100]. In this scenario, dynamic chromatin remodeling processes inducing histone modifications at promoters and distal regulatory elements and other epigenomic alterations would be the most likely molecular mechanism inducing anti-EGFR SR in our PDX models. Clearly, unidentified genetic aberrations such as noncoding regulatory mutations, and complex genetic/chromosomal changes including disruption of insulated neighborhood boundaries may also account for some of the observed transcriptional changes. Our in vivo models provide the unique platform not only to study the underlying molecular mechanisms driving transcriptional reprogramming but also allows the search for vulnerabilities of the cancer cells to interfere with its resistance evolutionary path, ultimately leading to more sustained treatment success.

### Limitations

A difference between patient SR tumors and our PDX model is the fact that 9 of our 12 SR models were derived from patients with UICC Stage I-III tumors, who do not receive anti-EGFR treatment in the clinical setting. Activated EGFR pathway signaling is known to support the growth of a subgroup of CRCs independent of their clinical stage and therefore more important for establishing the primary cancer growth and less critical for the development of the metastatic disease. This notion is also supported by our observation of response towards anti-EGFR therapy in earlier tumor stage CRC PDX models. Therefore, anti-EGFR SR mechanisms are likely stage independent and the transcriptional SR mechanism discovered in our study in non-stage IV models should also translate into later stage CRCs. Another limitation of our study is the fact that none of the patients included received anti-EGFR treatment throughout the duration of the study, precluding a direct comparison of the data from the PDX with the patient’s tumor progressing under treatment. However, Woolston et al., who specifically analyzed metastatic CRC lesions progressing under anti-EGFR monotherapy recently published that they were, in agreement with our data, unable to find mutations driving resistance in 64% of their biopsies [14]. Importantly, they used ultra-deep sequencing and included a substantial number of biopsies taken from lesions, which more than doubled their volume relative to their volume at the time of best response. This should have considerably reduced the risk that sequencing sensitivity or biopsy sampling errors are responsible for the detection failure of resistance mutations. Therefore, we are confident that our data is a true reflection of what can be found in the patient’s tumor.

## Conclusions

In summary, we demonstrate that PDX models are a suitable tool to efficiently establish SR tumors. They develop intertumoral heterogenic genetic and non-genetic anti-EGFR SR mechanisms comparable to what is observed in human CRC. We show that transcriptional reprogramming is an important SR mechanism in CRC PDX models under chronic anti-EGFR treatment. While non-genetic resistance mechanisms, including transcriptional reprogramming, are increasingly being recognized in several cancer types [101], they are currently largely ignored in the context of anti-EGFR SR in CRC. Our data emphasize the need for analyses of SR tumor tissues at a multi-omics level for a more differentiated molecular understanding of anti-EGFR SR in CRC. Lastly, identified non-genetic SR mechanisms could be addressed by targeted treatment, proving that SR PDX models are a valid tool enabling iterative treatment optimization targeting SR mechanisms, not possible in the patient. Ultimately, this should help to tailor more informed treatment strategies addressing anti-EGFR SR in patients.

## Supplementary Information

**Additional file 1: Table S1.** TruSeq Amplicon - Cancer Panel (Illumina).

**Additional file 2: Fig S1-S13.** Pdf file with all supplementary figures (Fig. S1-S13) with corresponding figure legends.

**Additional file 3: Table S2.** Growth curves of all chronically cetuximab treated tumors, GSEA snapshots of enrichment results, and summary of gene expression data for each individual xenograft tumor analyzed.

**Additional file 4: Table S3.** Tables of SNVs identified in primary resistant PDX models.

**Additional file 5: Table S4.** Summary of CGI predicted driver mutations and tables of SNVs identified in secondary resistant PDX models.

**Additional file 6: Table S5.** Selected gene sets enriched in phenotype “secondary resistant” (p ≤ 0.05).

**Additional file 7: Table S6.** Selected genes with potential function in SR for the 10 models tested.

## Data Availability

Exome sequencing has been deposited into the NCBI BioProject database under the BioProject ID PRJNA596887 (https://www.ncbi.nlm.nih.gov/bioproject/?term=PRJNA596887) [31]. The RNA sequencing and Agilent array data have been deposited in the NCBI’s Gene Expression Omnibus (GEO) database (accession number GSE141861(https://www.ncbi.nlm.nih.gov/geo/query/acc.cgi?acc=GSE141861) [37] and GSE140973 (https://www.ncbi.nlm.nih.gov/geo/query/acc.cgi?acc=GSE140973) [46], respectively). Exome sequencing used for CNV analyses has been deposited into the European Genome-phenome Archive (EGA) database under the EGA study ID EGAS00001005320 (https://ega-archive.org/studies/EGAS00001005320) [41].

## Abbreviations

CET: Cetuximab
CRC: Colorectal cancer
ctDNA: Circulating tumor DNA
GF: Growth factor
EGFR: Epidermal growth factor receptor
mABs: Monoclonal antibodies
PDX: Patient-derived xenograft
SR: Secondary resistance
wt: Wildtype
