# Supplementary material for: Secondary resistance to anti-EGFR therapy by transcriptional reprogramming in patient-derived colorectal cancer models
Source: Genome Med. 2021 Jul 16;13:116. doi: 10.1186/s13073-021-00926-7 (PMC8283888; doi:10.1186/s13073-021-00926-7)
Supplement: Supplementary file 2 — Additional file 2: Fig S1-S13. Pdf file with all supplementary figures (Fig. S1-S13) with corresponding figure legends. [file 13073_2021_926_MOESM2_ESM.pdf]

Additional file 2: Fig. S1-S13

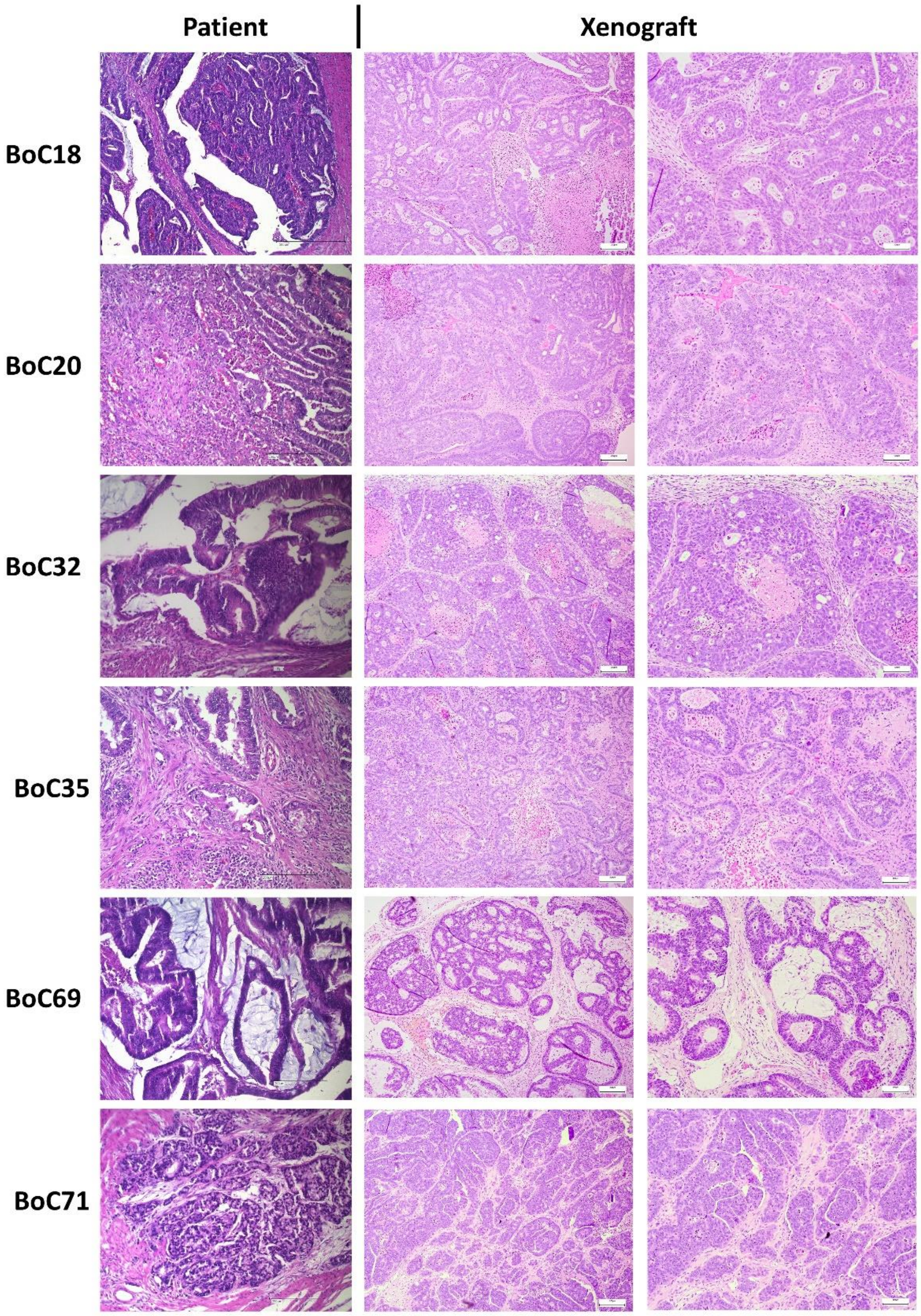

Fig. S1 Xenografted tumors retained the histopathologic characteristics of original samples. Hematoxylin and eosin stains of representative cases are shown.

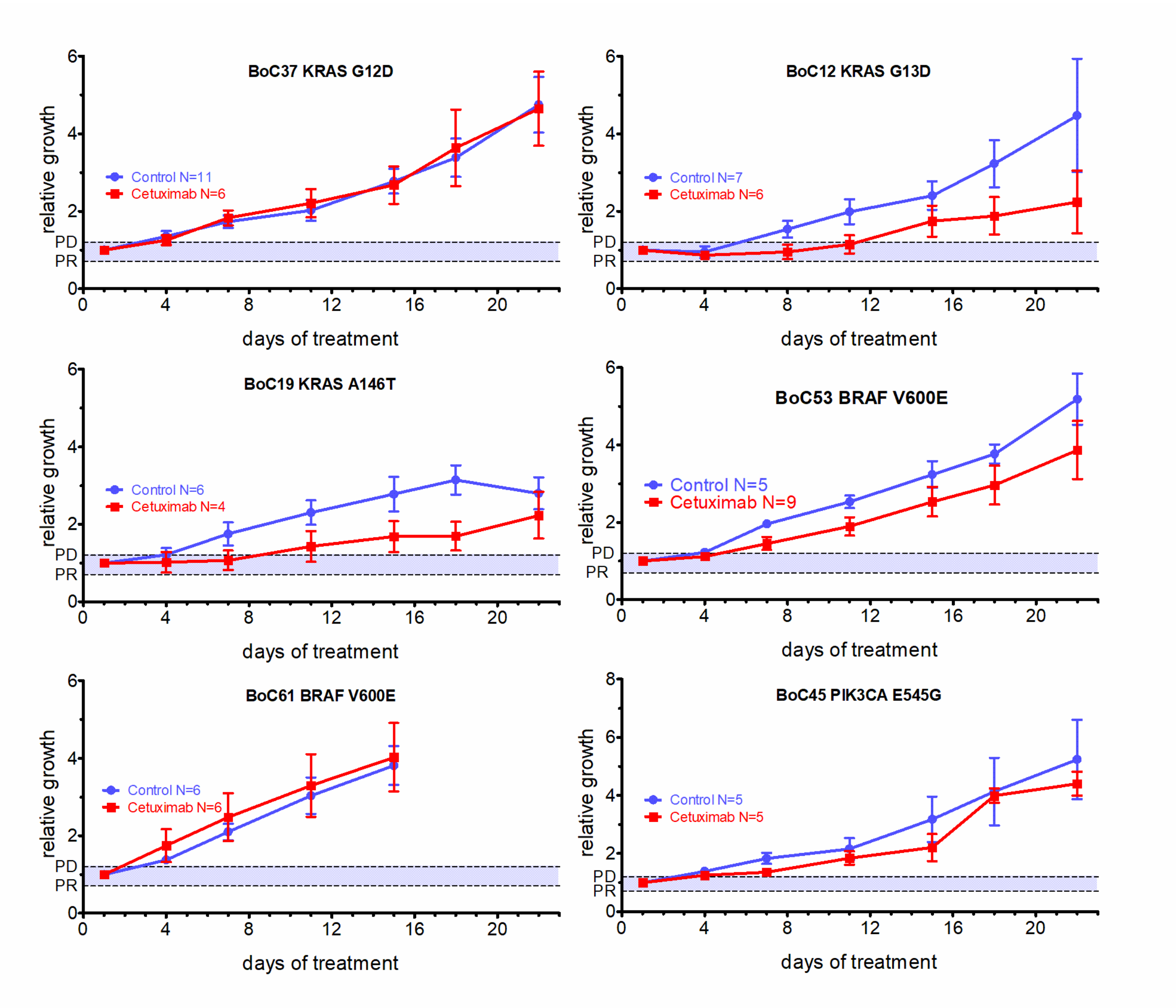

Fig. S2 Primary response pattern of additional PDX models with primary resistance (activating mutation in *KRAS*, *BRAF*, or *PIK3CA*). Relative growth curves are derived from mean values ± SEM (error bars). PD, progressive disease; PR, partial response.

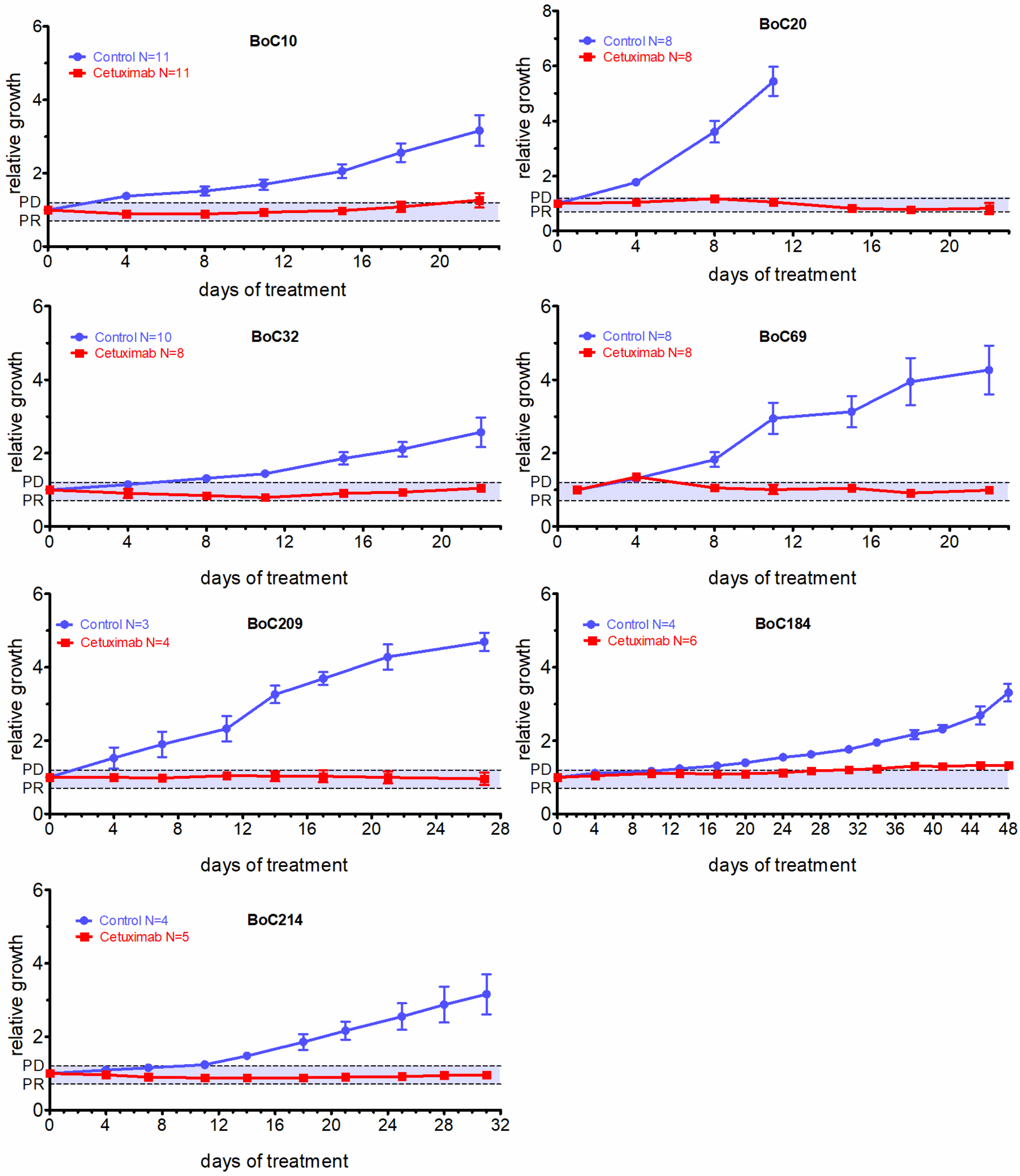

Fig. S3 Primary response pattern of additional PDX models with stable disease (*KRAS*, *NRAS*, *BRAF*, and *PIK3CA* wt). Relative growth curves are derived from mean values ± SEM (error bars). PD, progressive disease; PR, partial response.

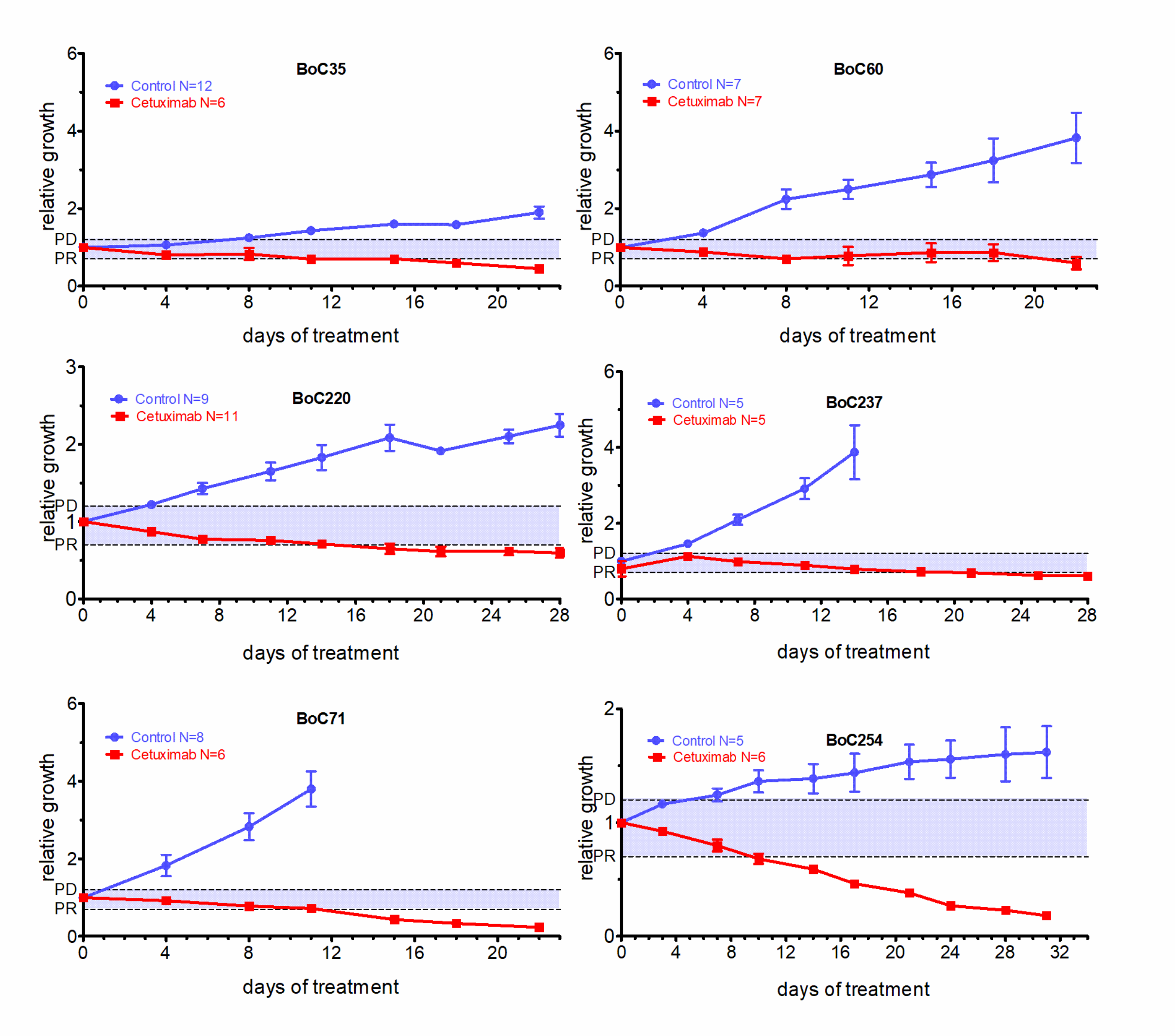

Fig. S4 Primary response pattern of additional PDX models with partial or complete response (*KRAS*, *NRAS*, *BRAF*, and *PIK3CA* wt). Relative growth curves are derived from mean values ± SEM (error bars). BoC220 and 254, treatment starting volume was 400mm^3. PD, progressive disease; PR, partial response.

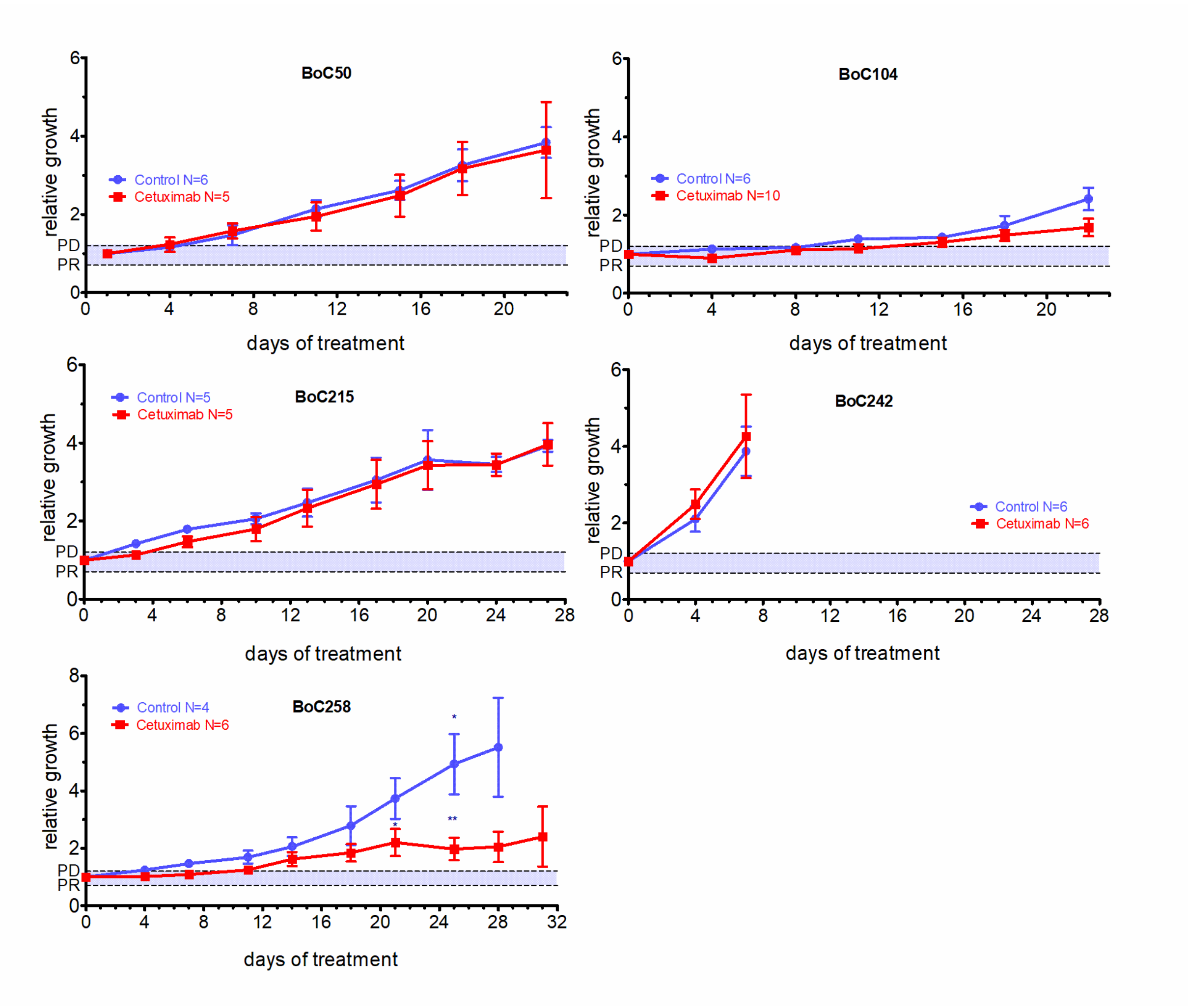

Fig. S5 Primary response pattern of additional PDX models with primary resistance (*KRAS*, *NRAS*, *BRAF*, and *PIK3CA* wt). Relative growth curves are derived from mean values ± SEM (error bars).*, each asterisk represents a tumor that was taken out of the treatment cohort at the indicated time point either because the tumor reached the maximum size criteria or due to health issues of the animal. PD, progressive disease; PR, partial response.

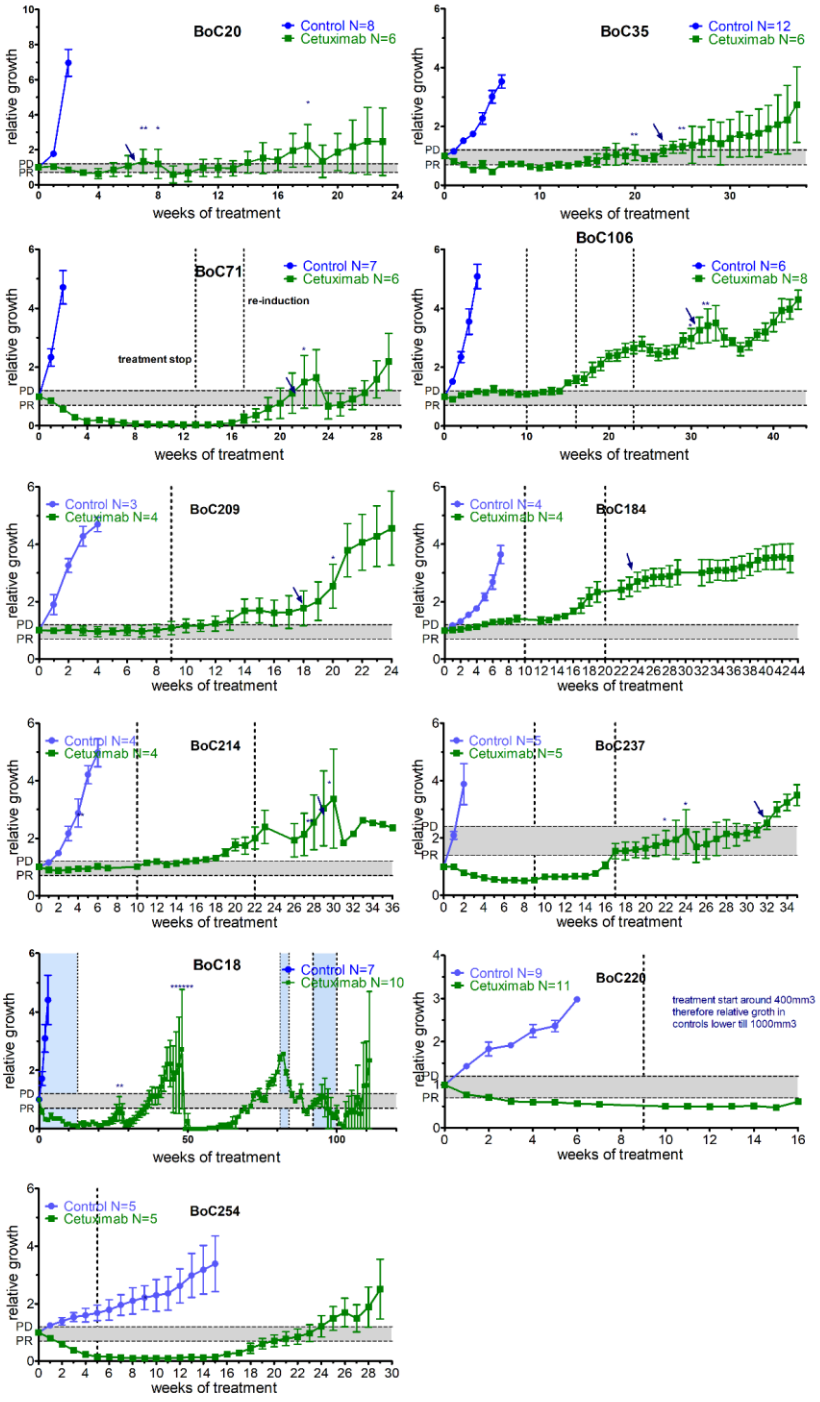

Fig. S6 Anti-EGFR secondary resistant PDX models can be generated with high efficiency. The growth pattern of PDX models not shown in Figure. 2B with long-term cetuximab treatment and initial treatment response to anti-EGFR therapy. For models BoC20, 35, and 71, time to progression was defined by the mean growth curve crossing the PD borderline. In Boc106 treatment was discontinued for 3 out of 4 animals (6 of 8 tumors) between weeks 10 and 16 (dotted lines); for one animal treatment was continued in sub-therapeutic dose (1:100 of starting dose). Between week 16-23 treatment was continued in all animals in sub-therapeutic dose (1:100 or 1:10; dotted lines) and from week 24 onwards all animals received full dosage. This led to growth stabilization for another 9 weeks until secondary resistance could finally be detected during week 31 (tumor volume increased by 20% relative to volume measured in week 24). In BoC209, 184, 214, and 237 treatment was paused at the first dotted line. Animals were re-treated as soon as at least one tumor per animal reached a volume of approx. 400-500mm^3.

BoC18 showed complete response and did not develop secondary resistance even after more than 600 days of intermittent treatment (periods of standard cetuximab treatment are shown as light blue shades areas). In BoC220 and BoC254 treatment was paused at week 5 and 9, respectively, following initial response. These models are still awaiting to reach 500mm^3 to reinitiate treatment. Arrows indicate the time point progressive disease was observed. PD, progressive disease; PR, partial response; gray shades area, stable disease; Relative growth curves are derived from mean values ± SEM (error bars). *, each star represents a tumor that was taken out of the treatment cohort at the indicated time point. Please note that this can lead to marked changes in mean tumor volumes (see also Additional file 4: Table S3 for individual growth curves)

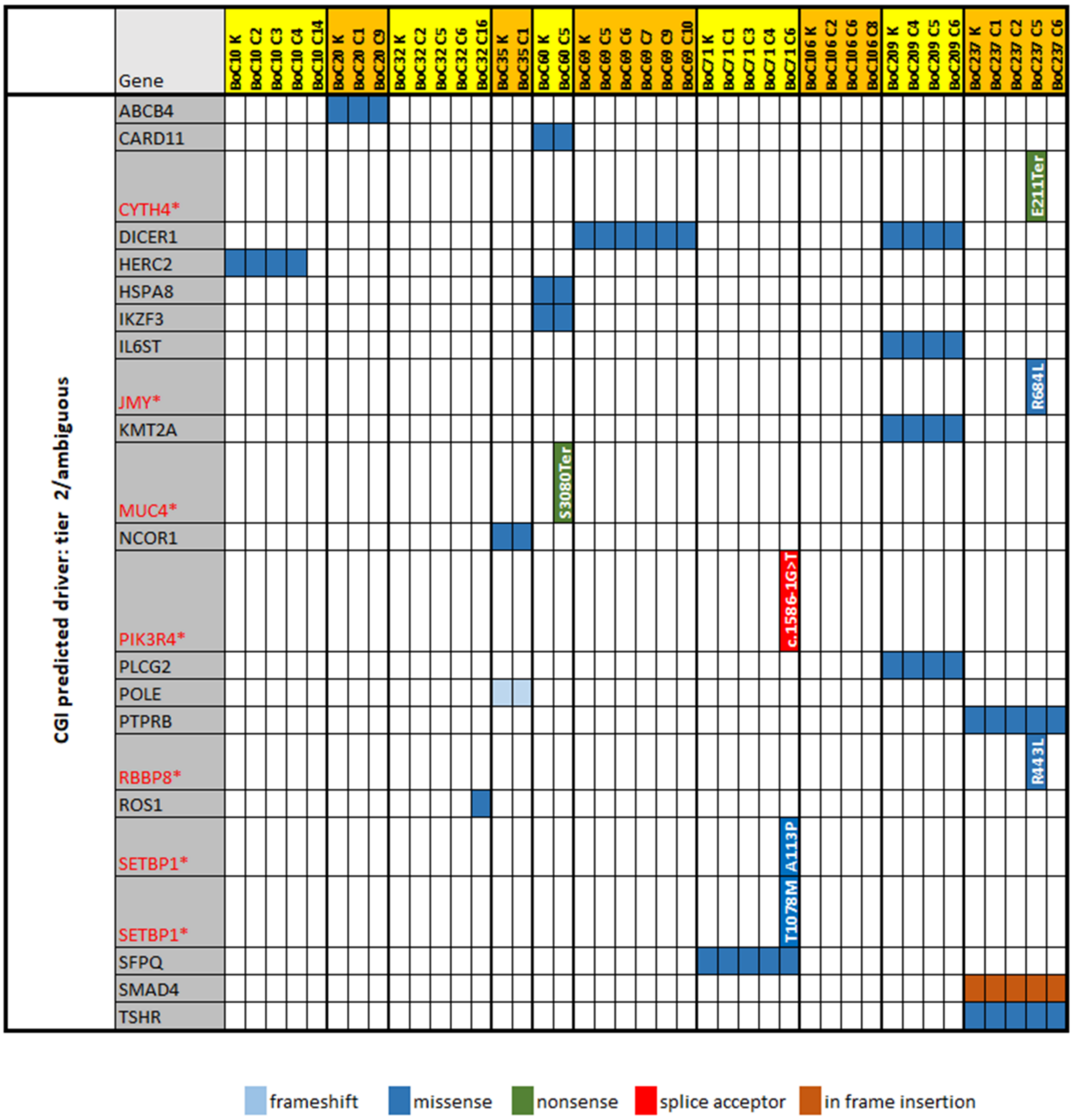

Fig. S7 Additional genomic alterations identified in SR PDX models. Cancer gene mutations with unclear functional relevance identified in the 10 PDX models. Acquired mutations in the SR models are indicated by an asterisk. K, untreated control tumor; C, cetuximab SR tumor.

Pathway	PDX Model	BoC71					BoC35			BoC60			BoC237			
	Response	CR					PR									
	Treatment status	BL	ST	SR			BL	ST	SR	BL	ST	SR	BL	ST	SR	
	CRIS Class	CRIS-B	CRIS-E	CRIS-E	CRIS-E	CRIS-B	CRIS-B	CRIS-C	CRIS-C	CRIS-C	CRIS-C	CRIS-E	CRIS-E	CRIS-C	CRIS-C	CRIS-A
Pathway	Gene Symbol	BoC71K	BoC715dC	BoC71C	BoC71C-MEK2 mut	BoC71C-KRAS mut	BoC35K	BoC355dC	BoC35C	BoC60K	BoC605dC	BoC60C EGFR ECD mut	BoC237K	BoC2375dC	BoC237C	BoC237C-MEK1 mut
ERBB	AREG	16	14	15	14	15	14	14	15	16	14	16	15	14	12	14
	EREG	14	13	13	12	13	10	9	10	12	11	12	11	10	8	10
	TGFA	11	11	11	10	10	11	11	10	11	11	11	10	10	10	10
	HBEGF	9	8	9	8	9	8	8	8	8	8	8	7	5	6	8
	EGF	8	8	7	9	7	6	6	8	7	10	8	8	8	8	8
	NRG1	3	3	3	2	3	3	3	3	3	4	3	3	3	4	3
	NRG2	5	6	4	4	4	5	5	5	4	4	5	4	4	5	4
	NRG3	2	2	2	3	2	2	2	2	2	3	2	3	3	2	4
	NRG4	4	2	4	3	3	4	3	3	3	2	4	4	4	4	4
	EGFR	11	10	10	11	11	10	10	11	10	11	10	10	10	10	11
	ERBB2	13	14	13	13	14	14	14	13	12	13	13	13	14	14	13
	ERBB3	13	14	13	13	13	13	14	13	13	14	13	13	13	13	13
	ERBB4	2	4	2	2	2	3	3	3	2	2	2	2	2	2	2
MET	MET	14	13	13	13	13	14	14	14	14	14	15	14	14	13	14
	HGF	3	3	2	2	2	3	3	3	2	3	3	4	4	2	2
FGFR	FGF3	7	7	7	7	7	9	8	9	6	6	6	6	7	7	7
	FGF9	5	5	7	6	6	5	7	6	6	7	7	5	6	7	4
	FGF13	6	8	8	7	8	7	8	7	4	5	5	8	9	8	8
	FGF19	2	2	2	2	3	2	2	2	3	2	4	2	2	2	2
	FGF20	9	9	7	5	9	4	4	5	2	3	4	2	2	2	2
	FGFR1	4	5	4	2	4	5	6	7	4	4	5	2	4	9	4
	FGFR2	6	6	6	6	6	9	9	10	8	8	7	9	11	11	10
	FGFR3	10	9	10	11	10	12	12	12	14	14	13	12	12	11	12
	FGFR4	9	8	9	8	8	10	9	9	10	10	10	9	9	8	9
IGFR	IGF1	3	4	5	5	3	3	4	4	4	4	5	3	3	4	3
	IGF2	5	3	11	5	7	5	3	5	5	5	4	3	2	2	4
	IGF1R	10	11	10	10	11	11	12	12	11	11	11	11	11	11	11
PDGFR	PDGFA	10	11	12	10	10	11	10	10	10	11	11	10	12	13	11
	PDGFB	6	5	5	6	7	9	8	9	7	7	7	7	6	6	6
	PDGFC	2	2	2	2	6	3	2	3	3	4	4	3	4	4	2
	PDGFRA	7	9	7	7	8	8	9	9	8	9	10	7	8	8	7
	PDGFRB	3	3	3	3	4	2	3	2	4	3	4	4	4	3	3
KIT	KIT	4	4	3	3	4	7	6	8	5	5	3	4	4	6	3
	KITLG	10	10	9	10	10	9	9	9	11	12	10	11	10	10	10
RAS-RAF-MEK-ERK	KRAS	11	11	10	11	12	11	11	10	10	11	10	11	11	12	11
	NRAS	10	10	10	11	10	10	10	10	10	10	9	10	9	10	9
	HRAS	11	10	11	11	10	10	10	10	10	10	11	10	9	10	10
	BRAF	8	8	8	8	8	8	9	9	9	9	9	8	8	8	8
	RAF1	11	10	10	10	11	10	10	11	11	11	11	11	11	11	11
	MAPK1	11	10	11	11	11	9	9	10	9	9	9	10	9	10	10
	MAPK3	10	10	10	10	10	11	10	10	10	10	10	10	10	10	10
	MAP2K1	11	10	10	11	11	11	10	11	11	11	12	11	10	10	11
	MAP2K2	11	11	12	12	11	11	11	11	12	12	12	12	11	11	11
PI3K-AKT	PIK3CA	9	9	9	9	9	9	9	9	9	9	9	9	9	9	9
	PTEN	8	9	9	9	9	10	10	10	10	10	10	10	10	10	10
	AKT1	12	12	12	11	12	11	12	12	12	12	12	11	11	11	11
	AKT2	11	10	10	11	11	11	11	11	10	10	10	11	11	11	11
	AKT3	5	5	5	5	5	5	6	6	5	5	6	5	5	6	5

Pathway	PDX Model	BoC10			BoC20			BoC32			BoC69			BoC106				BoC209			
	Response	SD																			
	Treatment status	BL	ST	SR	BL	ST	SR	BL	ST	CR	BL	ST	CR	BL	ST	CR		BL	ST	CR	
	CRIS Class	CRIS-D	CRIS-D	CRIS-D	CRIS-C	CRIS-C	CRIS-C	CRIS-B	CRIS-C	CRIS-C	CRIS-D	CRIS-D	CRIS-C	CRIS-C	CRIS-C	CRIS-C	CRIS-C	CRIS-E	CRIS-E	CRIS-E	CRIS-E
Pathway	Gene Symbol	BoC10K	BoC105dC	BoC10C	BoC20K	BoC205dC	BoC20C	BoC32K	BoC325dC	BoC32C	BoC69K	BoC695dC	BoC69C	BoC106K	BoC1065dC	BoC106C	BoC106C-KRAS amp	BoC209K	BoC2095dC	BoC209C	BoC209C-KRAS mut
ERBB	AREG	14	13	13	15	15	14	14	14	14	15	15	15	13	13	13	14	14	15	14	15
	EREG	10	9	9	12	11	11	10	10	9	12	12	12	10	9	10	11	11	11	10	11
	TGFA	11	10	11	10	10	10	10	11	10	11	11	11	11	10	10	10	10	10	10	10
	HBEGF	8	6	6	8	6	7	7	7	7	9	8	8	7	7	7	7	9	9	8	9
	EGF	3	2	3	6	3	4	9	8	8	2	2	3	9	9	7	9	2	2	2	4
	NRG1	5	3	4	3	3	3	3	3	3	4	4	3	2	3	4	3	4	4	3	3
	NRG2	6	5	5	5	5	5	5	5	4	5	5	4	6	4	5	5	4	4	4	3
	NRG3	2	2	2	2	2	2	2	2	2	2	2	2	2	2	2	2	2	3	3	3
	NRG4	5	3	4	4	4	4	3	3	3	4	4	3	2	3	4	3	4	3	4	3
	EGFR	9	9	8	10	8	9	10	9	9	9	9	9	10	9	10	9	9	8	7	8
	ERBB2	13	13	13	13	13	13	13	13	14	13	13	13	13	14	14	14	13	13	13	12
	ERBB3	13	13	13	13	14	13	13	13	13	12	12	12	13	14	13	13	12	13	13	12
	ERBB4	4	2	3	3	3	2	2	3	3	3	2	3	2	2	2	2	2	3	2	2
MET	MET	14	14	13	14	14	13	14	14	13	14	14	14	14	14	13	14	13	14	14	13
	HGF	2	2	4	2	2	2	2	3	3	2	2	2	3	4	4	3	2	4	3	3
FGFR	FGF3	7	8	13	7	8	7	6	6	7	7	7	9	8	6	7	6	7	8	10	9
	FGF9	9	11	11	5	5	7	5	6	6	9	9	10	6	7	7	6	9	11	12	11
	FGF13	7	7	10	6	7	8	8	10	9	4	4	8	4	7	7	6	6	7	8	7
	FGF19	4	6	12	2	2	2	2	2	2	2	2	5	2	2	2	2	6	5	4	6
	FGF20	6	8	13	4	4	3	5	2	4	3	3	9	5	4	4	6	10	10	11	10
	FGFR1	6	7	7	4	6	5	6	4	6	8	8	9	7	7	6	7	4	4	9	6
	FGFR2	9	9	8	8	9	9	8	8	9	9	9	10	7	8	8	7	8	8	8	8
	FGFR3	15	15	15	12	13	13	12	12	12	12	12	12	11	12	13	12	12	13	12	12
	FGFR4	9	9	9	9	9	9	8	8	8	10	10	9	10	9	9	10	10	9	9	9
IGFR	IGF1	4	5	5	4	4	5	3	3	3	4	4	8	3	4	4	4	3	3	7	4
	IGF2	19	19	19	5	6	6	7	7	7	18	18	18	6	6	5	7	19	19	19	19
	IGF1R	10	10	11	11	12	11	10	11	11	9	10	10	11	11	11	10	8	8	6	7
PDGFR	PDGFA	10	11	12	10	12	13	8	10	10	9	9	11	10	10	11	10	11	12	12	11
	PDGFB	6	6	6	5	5	6	5	4	5	4	5	6	8	7	6	8	6	7	7	7
	PDGFC	10	11	11	2	3	2	2	2	2	9	9	10	2	2	3	2	8	9	11	10
	PDGFRA	9	10	12	6	7	9	8	10	10	8	9	8	8	9	11	9	7	9	10	8
	PDGFRB	5	5	4	3	3	3	3	2	3	3	4	3	2	4	4	4	5	5	4	6
KIT	KIT	8	7	6	7	2	6	10	7	7	9	8	9	7	6	6	5	3	3	5	2
	KITLG	11	10	11	10	9	10	9	10	10	10	9	10	9	9	10	9	10	10	9	9
RAS-RAF-MEK-ERK	KRAS	11	11	11	11	11	11	11	11	10	11	11	11	11	11	11	14	11	11	11	11
	NRAS	10	9	10	9	9	9	10	10	10	10	10	10	10	10	10	10	10	10	10	10
	HRAS	10	10	11	11	10	10	11	10	10	11	11	11	10	10	10	11	10	10	10	10
	BRAF	8	8	9	8	8	8	10	9	9	8	8	8	9	9	8	9	8	8	8	8
	RAF1	11	11	11	11	11	11	11	11	11	11	11	11	11	11	11	11	11	11	11	10
	MAPK1	9	9	9	9	9	9	10	9	9	10	10	10	9	9	9	9	10	10	9	10
	MAPK3	10	10	10	10	10	10	10	10	9	9	9	10	10	10	10	10	11	10	10	10
	MAP2K1	11	11	10	11	11	11	11	10	10	11	11	11	11	10	11	11	11	10	10	10
	MAP2K2	11	11	12	11	11	11	12	11	12	11	11	11	11	11	11	11	11	11	10	11
PI3K-AKT	PIK3CA	9	9	10	10	10	10	9	9	9	9	9	9	8	9	9	9	8	9	9	8
	PTEN	10	10	10	10	10	10	9	10	9	10	10	10	10	10	10	10	10	9	10	10
	AKT1	12	12	12	11	12	11	12	12	12	12	12	12	12	12	12	12	12	11	12	12
	AKT2	10	10	11	12	12	11	11	11	11	10	10	10	11	11	11	12	11	10	11	11
	AKT3	5	6	8	3	4	5	4	6	6	5	6	5	5	5	6	5	5	7	7	5

log₂ 5 — 9 — 18

Fig. S8 Transcriptomic subtypes and expression of growth factor signaling pathway genes. The upper part shows the CRIS subtypes of the 10 models linked to their CET treatment and response status as well as to driver alterations. BL, baseline tumor untreated; ST, CET sensitive tumors; SR, secondary resistant tumor. The lower part shows expression values for relevant genes linked to growth factor signaling. Shown are expression values for untreated control tumors (K), tumors treated for 5 days with CET (5dC), and tumors that developed SR under chronic CET treatment (C). SR tumors harboring driver alterations are given in a separate column. mut, mutation in the indicated gene; amp, amplification in the indicated gene. Data shown as quantile normalized log2 values. Log2 expression values below 5 are considered background.

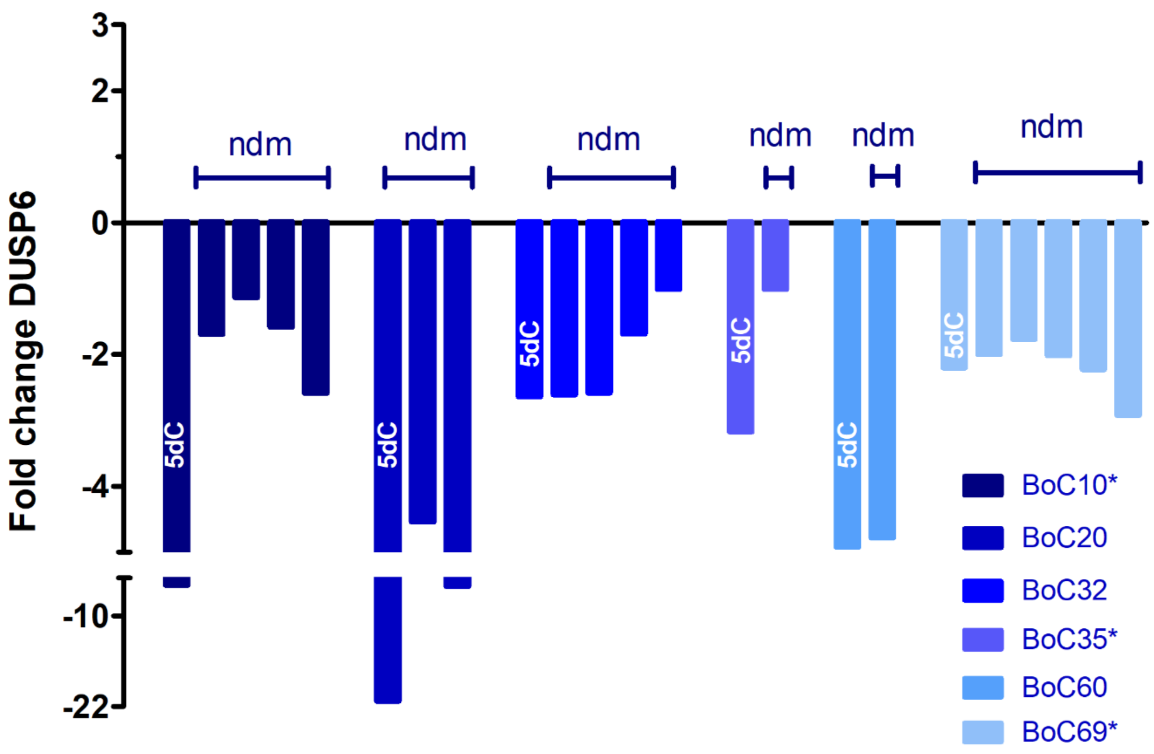

Fig. S9 DUSP6 expression in SR PDX tumors without driver gene mutations. Fold changes are given relative to untreated control tumors in SR PDX models compared to the corresponding cetuximab sensitive tumors treated for 5 days (5dC). Fold changes are calculated as mean values from at least three measurements. *, models with KRAS_SIGNALLING_UP" set found to be enriched, ndm, no driver mutation.

PDX Model	BoC10				BoC20		BoC32				BoC 35	BoC 60	BoC69					BoC71				BoC106			BoC209			BoC237		
Gene	C2/5dC	C3/5dC	C4/5dC	C14/5dC	C1/5dC	C9/5dC	C2/5dC	C5/5dC	C6/5dC	C16/5dC	C1/5dC	C5/5dC*	C5/5dC	C6/5dC	C7/5dC	C9/5dC	C10/5dC	C1/5dC*	C3/5dC*	C4/5dC*	C6/5dC	C2/5dC*	C6/5dC	C8/5dC	C4/5dC	C5/5dC*	C6/5dC	C1/5dC*	C2/5dC	C5/5dC
ALDH1A1 (S)													3.4	3.1	3.1	4.8	4.2							2.7						2.2
ALDH1A1 (A)													6.2	5.5	6.3	6.1	5.1							8.4						2.6
ALDH1A3 (S)		3.3	2.2					2.4	4.9	2.7		12						2.0						6.5						
ALDH1A3 (A)		5.5	3.1					3.3	4.8	7.2		5.8						2.0						12						
BAMBI (S)	12	23	15	5.0				4.8	4.0			24	2.3			6.8	4.8				2.4									
BAMBI (A)	52	130	69	33				4.8	3.9			30	2.9			5.4	5.0				2.5									
CXCR4 (S)		9.0			5.1	10					6.6		2.6			2.2	2.4	6.5		2.8	10			25		2.5		3.2	2.6	2.9
CXCR4 (A)		23			12	24					12		3.5			3.7	3.6	8.7		6.7	17					2.3		11	5.1	8.6
DEFA5 (S)					536	502	24	52	15		2.2		32	36	40	37	61	6.1			13	10	78	210						
DEFA5 (A)					4159	1807	44	11	22		18		187	151	281	254	406	3.2			9.3									
DEFA6 (S)					82	110		3.4			10	17	26	25	33	29	44				3.4		4.3	84						
DEFA6 (A)					842	665		10			4.7	15	101	68	137	106	140				2.6		21	737						
DLL1 (S)					23	14		2.1					17	9.3	21	4.6	8.4													
DLL1 (A)					81	39		3.4					5.3	4.2	5.5	4.8	4.4													
FGF18 (S)	3.8	11	7.1																											
FGF18 (A)	9.4	11	7.2																											
FGF19 (S)	56	73	58	16												2.4	3.2													
FGF19 (A)	98	173	97	51												25	24													
FGF20 (S)	35	48	38	12				3.7					19	11	16	20	22								4.9					
FGF20 (A)	65	114	73	50				18					35	46	59	74	103								2.1					
FGF3 (S)	3.1										n.d.		n.d.	n.d.	n.d.	n.d.	n.d.									3.3				
FGF3 (A)	35										4		3	3	3	5	5									2.3				
FGF9 (S)					10										2.5		2.3		19		17									10
FGF9 (A)					4.6										2.0		2.1		2.4		2.3									4.2
FGFR1 (S)	2.0	12			5.1		6.0	4.4	10	4.5	7.8														16	6	22		42	25
FGFR1 (A)	3.1	2.1			2.0		2.2	2.2	4.4	2.4	3.8														12	3.2	34		35	18
FGFR2 (S)								2.7			3.4				2.3				3.8		6.9									
FGFR2 (A)								2.1			2.5				2.4				2.2		2									
IGF1 (S)													12	16	19	17	29	3.3							35		33			
IGF1 (A)													15	15	29	25	50	2.4							15.4		10			
IGF2 (S)																					14									
IGF2 (A)																					345.0									
IRX3 (S)							112	89	71		9.0		41	41	62	24	48					10	10	4.1	2.1				8.7	8.6
IRX3 (A)							17	10	11		6.6		17	17	21	16	20					12	11	6	2.5				46	47
IRX5 (S)						14	4.2	6.9	4.9	7.6			11	19	14	9	16													
IRX5 (A)						8.7	3.4	5.4	2.9	4.6			10	11	11	10	12													
KIT (S)					7.7	14					4.5					2.4								11						
KIT (A)					2.82	2.1					3.9					3.3								3						
OLFM4 (S)					80	86	10	14	6.2			47											3.3	10						
OLFM4 (A)					362	228	23	36	7.3			383											14	605						
PDGFA (S)													5.9	4.9	5.9	6.0	6.4													
PDGFA (A)													7.4	7.4	7.7	7.7	8.4													
PLA2G4A (S)	71	216	90	21																										
PLA2G4A (A)	325	879	366	216																										
RASGRF1 (S)												9		4	5	4	7							2						
RASGRF1 (A)												13.86		2.78	4.05	2.74	4.63							2.01						
HOXA5 (S)												0.04																		
HOXA5 (A)												0.07																		
RASAL1 (S)					0.43								0.24	0.05	0.14	0.05	0.18													
RASAL1 (A)					0.33								0.19	0.14	0.18	0.15	0.19													

Fig. S10 Synopsis of confirmed candidate genes with a potential role in secondary resistance. Shown are fold changes in gene expression for the indicated genes derived from Agilent array data (A) and the corresponding fold changes detected via 3`RNA Seq (S) for individual SR tumors (C) relative to their expression in 5 days CET treated controls (5dC). n.d., not detected.

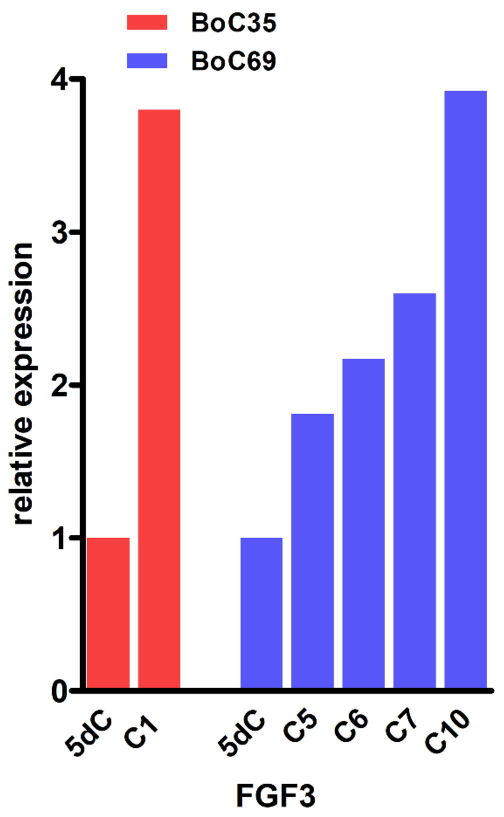

Fig. S11 qRT-PCR data confirming the overexpression of FGF3 in the indicated SR tumors. Relative FGF3 expression of the SR tumor (C) to the corresponding expression in the control tumor (5dC) normalized to either COX6C (BoC35) or GAPDH (BoC69).

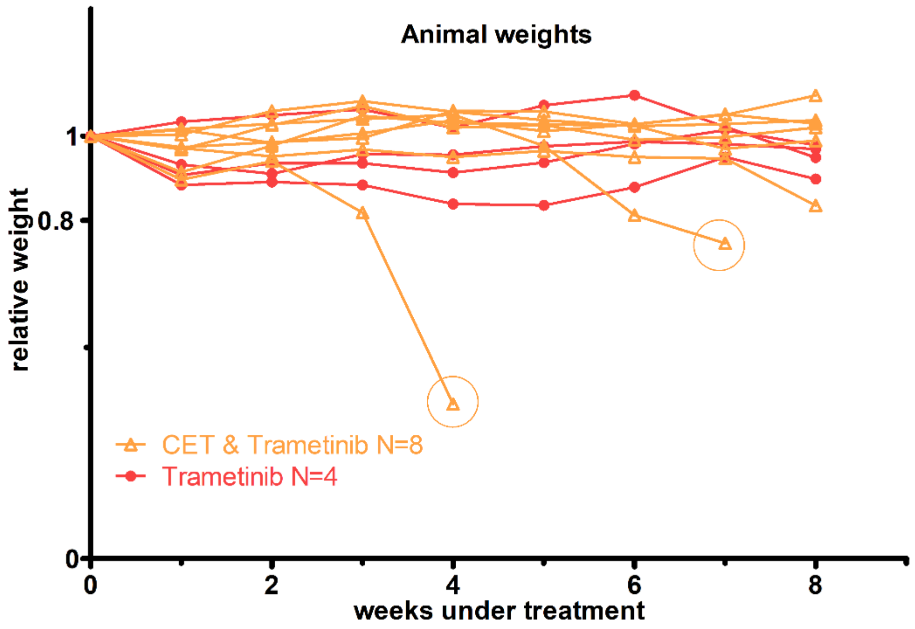

Fig. S12 Course of body weights for animals treated with Trametinib mono- or Trametinib-CET combination therapy. Shown are body weights relative to the weights of the animals at treatment start corresponding to the experiments shown in Fig. 9a and c. Critical losses are highlighted with circles.

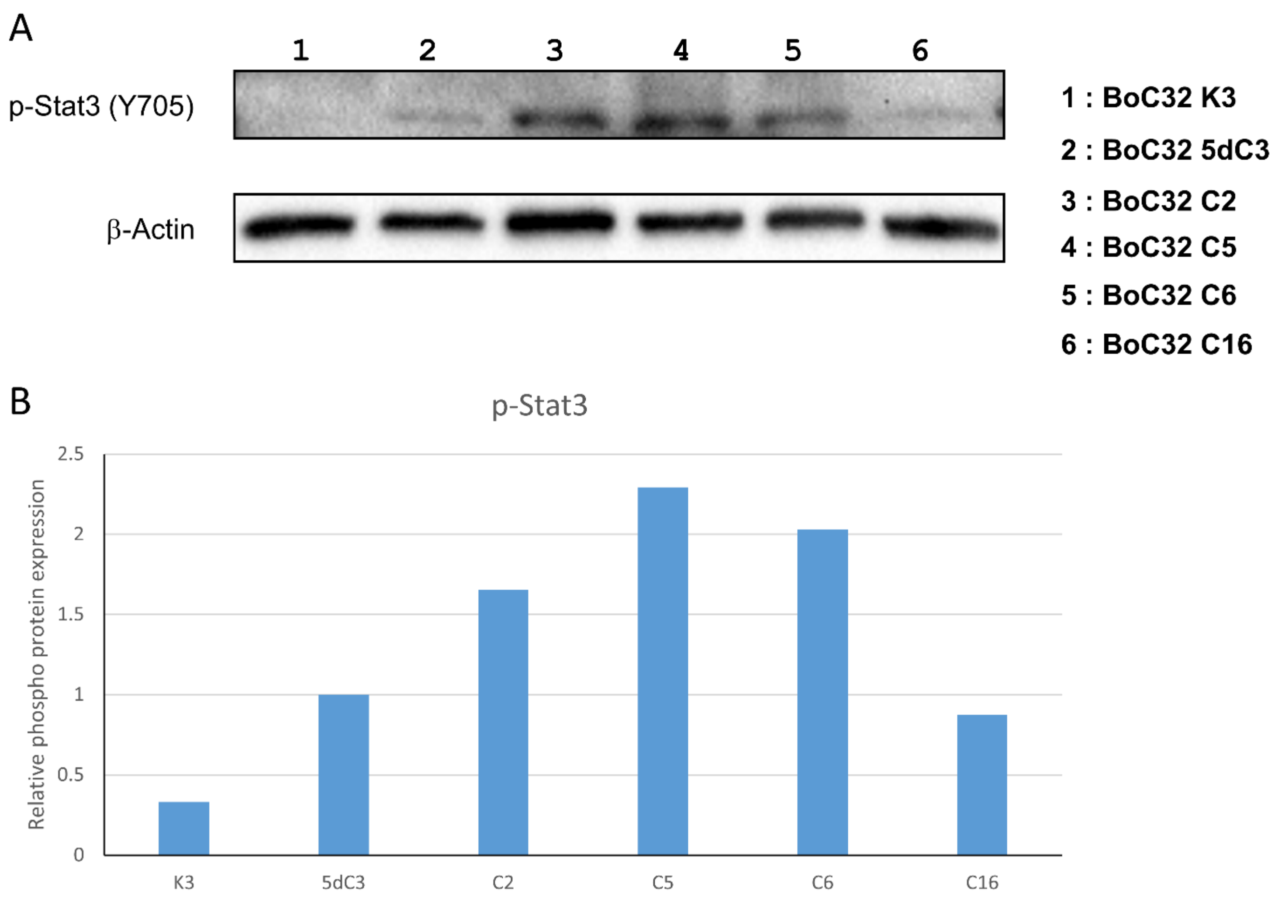

Fig. S13 Targeted treatment addressing SR. **a** pSTAT3 protein expression at Y705 [pSTAT3 (Y705)] in an untreated BoC32 tumor (K), a tumor treated for 5 days with cetuximab (5dC) and tumors which developed secondary resistance under chronic cetuximab treatment (C). **b** Relative quantification of pSTAT3 signal intensities to the pSTAT3 intensity of the 5dC tumor normalized with the corresponding beta-actin signal intensities using Image Lab (BioRad).
